# Structural variant allelic heterogeneity in *MECP2* duplication syndrome provides insight into clinical severity and variability of disease expression

**DOI:** 10.1186/s13073-024-01411-7

**Published:** 2024-12-18

**Authors:** Davut Pehlivan, Jesse D. Bengtsson, Sameer S. Bajikar, Christopher M. Grochowski, Ming Yin Lun, Mira Gandhi, Angad Jolly, Alexander J. Trostle, Holly K. Harris, Bernhard Suter, Sukru Aras, Melissa B. Ramocki, Haowei Du, Michele G. Mehaffey, KyungHee Park, Ellen Wilkey, Cemal Karakas, Jesper J. Eisfeldt, Maria Pettersson, Lynn Liu, Marwan S. Shinawi, Virginia E. Kimonis, Wojciech Wiszniewski, Kyle Mckenzie, Timo Roser, Angela M. Vianna-Morgante, Alberto S. Cornier, Ahmed Abdelmoity, James P. Hwang, Shalini N. Jhangiani, Donna M. Muzny, Tadahiro Mitani, Kazuhiro Muramatsu, Shin Nabatame, Daniel G. Glaze, Jawid M. Fatih, Richard A. Gibbs, Zhandong Liu, Anna Lindstrand, Fritz J. Sedlazeck, James R. Lupski, Huda Y. Zoghbi, Claudia M. B. Carvalho

**Affiliations:** 1 Department of Pediatrics, Section of Neurology and Developmental Neuroscience, Baylor College of Medicine, Houston, TX 77030 USA; 2 Department of Molecular and Human Genetics, Baylor College of Medicine, Houston, TX 77030 USA; 3 Department of Pediatrics, Baylor College of Medicine, Houston, TX 77030 USA; 4 Jan and Dan Duncan Neurological Research Institute at Texas Children’s Hospital, Houston, TX 77030 USA; 5 Pacific Northwest Research Institute, Seattle, WA 98122 USA; 6 Human Genome Sequencing Center, Baylor College of Medicine, Houston, TX 77030 USA; 7 The Meyer Center for Developmental Pediatrics and Autism, 8080 North Stadium Drive, Houston, TX 77054 USA; 8 University Otolaryngology, East Greenwich, RI 02818 USA; 9 Department of Pediatrics, Division of Neurology, University of Louisville, Louisville, KY 40202 USA; 10 Department of Molecular Medicine and Surgery, Karolinska Institutet, Stockholm, Sweden; 11 Department of Neurology, Division of Epilepsy, University of North Carolina, Chapel Hill, NC 27599 USA; 12 Department of Pediatrics, Division of Genetics and Genomic Medicine, Washington University School of Medicine, St. Louis, MO 63110 USA; 13 Department of Pediatrics, Division of Genetics and Genomic Medicine, University of California, Irvine, CA 92697 USA; 14 Department of Molecular and Medical Genetics, Oregon Health and Science University, Portland, OR 97239 USA; 15 Department of Pediatrics, Division of General and Community Pediatrics, University of Alberta, Edmonton, AB T6G 2R7 Canada; 16 Department of Pediatrics, Division of Pediatric Neurology, Developmental Medicine and Social Pediatrics, Dr. Von Haunersches Children’s Hospital, Ludwig Maximilian University of Munich, Munich, 80337 Germany; 17 Department of Genetics and Evolutionary Biology, Institute of Biosciences, São Paulo - SP, 05508-090 Brazil; 18 Department of Genetics, San Jorge Children’s Hospital, San Juan, 00771 Puerto Rico; 19 Division of Neurology, Department of Pediatrics, Children’s Mercy Kansas City, Kansas City, MO 64108 USA; 20 Department of Pediatrics, Jichi Medical University, Shimotsuke-City, Tochigi, 329-0498 Japan; 21 Department of Pediatrics, Osaka University Graduate School of Medicine, Osaka, 565-0871 Japan; 22 Texas Children’s Hospital, Houston, TX 77030 USA; 23 Howard Hughes Medical Institute and Jan and Dan Duncan Neurological Research Institute, Houston, TX 77030 USA

**Keywords:** *MECP2* duplication syndrome, MRXSL, Tandem duplication, Terminal duplication, Clinical severity, Survival

## Abstract

**Background:**

*MECP2* Duplication Syndrome, also known as X-linked intellectual developmental disorder Lubs type (MRXSL; MIM: 300260), is a neurodevelopmental disorder caused by copy number gains spanning *MECP2*. Despite varying genomic rearrangement structures, including duplications and triplications, and a wide range of duplication sizes, no clear correlation exists between DNA rearrangement and clinical features. We had previously demonstrated that up to 38% of MRXSL families are characterized by complex genomic rearrangements (CGRs) of intermediate complexity (2 ≤ copy number variant breakpoints < 5), yet the impact of these genomic structures on regulation of gene expression and phenotypic manifestations have not been investigated.

**Methods:**

To study the role of the genomic rearrangement structures on an individual’s clinical phenotypic variability, we employed a comprehensive genomics, transcriptomics, and deep phenotyping analysis approach on 137 individuals affected by MRXSL. Genomic structural information was correlated with transcriptomic and quantitative phenotypic analysis using Human Phenotype Ontology (HPO) semantic similarity scores.

**Results:**

Duplication sizes in the cohort ranging from 64.6 kb to 16.5 Mb were classified into four categories comprising of tandem duplications (48%), terminal duplications (22%), inverted triplications (20%), and other CGRs (10%). Most of the terminal duplication structures consist of translocations (65%) followed by recombinant chromosomes (23%). Notably, 65% of de novo events occurred in the Terminal duplication group in contrast with 17% observed in Tandem duplications. RNA-seq data from lymphoblastoid cell lines indicated that the *MECP2* transcript quantity in *MECP2* triplications is statistically different from all duplications, but not between other classes of genomic structures. We also observed a significant (*p* < 0.05) correlation (Pearson *R* = 0.6, Spearman *p* = 0.63) between the log-transformed *MECP2* RNA levels and MECP2 protein levels, demonstrating that genomic aberrations spanning *MECP2* lead to altered *MECP2* RNA and MECP2 protein levels. Genotype–phenotype analyses indicated a gradual worsening of phenotypic features, including overall survival, developmental levels, microcephaly, epilepsy, and genitourinary/eye abnormalities in the following order: Tandem duplications, Other complex duplications, Terminal duplications/Translocations, and Triplications encompassing *MECP2*.

**Conclusion:**

In aggregate, this combined analysis uncovers an interplay between *MECP2* dosage, genomic rearrangement structure and phenotypic traits. Whereas the level of MECP2 is a key determinant of the phenotype, the DNA rearrangement structure can contribute to clinical severity and disease expression variability. Employing this type of analytical approach will advance our understanding of the impact of genomic rearrangements on genomic disorders and may help guide more targeted therapeutic approaches.

**Supplementary Information:**

The online version contains supplementary material available at 10.1186/s13073-024-01411-7.

## Introduction

*MECP2 *maps to chromosome Xq28, includes four exons, and encodes the methyl CpG binding protein 2 (MECP2) (NM_004992.4). The protein fine tunes the expression of thousands of genes in the brain by binding to methylated cytosines predominantly in the CG and CAC context [1–3]. Loss-of-function variants, including exonic deletions and indels, of *MECP2 *cause Rett syndrome (MIM: 312750) [4], a severe postnatal neurodevelopmental disorder that primarily affects females. Conversely, copy number gains encompassing *MECP2 *lead to MRXSL (MIM: 300260), an X-linked genomic disorder primarily affecting males, with an estimated prevalence of one in 100,000 live male births [5].

The clinical presentation among MRXSL individuals is variable, but is characterized by core features that include infantile hypotonia, severe developmental delay/intellectual disability (DD/ID), poor or absent speech, progressive spasticity, gastrointestinal problems, frequent respiratory infections and epilepsy [6–13]. As more individuals with MRXSL are ascertained and investigated, further clinical features of the disorder are revealed [7, 9, 13]. For example, Miguet et al. studied 59 French male subjects and identified similar facial dysmorphia such as midface hypoplasia, open and small mouth, drooling, and tapered fingers in 93% of the patients. [7] Eye abnormalities (strabismus in 76% and hypermetropia in 54%) and decreased pain sensitivity (78%) are additional common features, and pulmonary hypertension (2/11, 18%) contribute to the early death in this population [7]. Peters et al. examined 48 individuals from the USA including 43 males and 5 females in whom irritability (58%), sleep disturbances (43.8%) and breathing abnormalities (25%) were newly identified features [13].

A few studies investigated the molecular basis of clinical variability and severity, but these did not identify underlying molecular causes [7, 9, 14], although some potential clues began to emerge. Peters et al. observed that patients who harbor duplications that include the gene *RAB39B *have a higher Rett syndrome-based clinical severity score [13]. Pascual-Alonso et al. studied 19 probands, three with *MECP2* duplications resulting from unbalanced translocations to autosomes that were proposed to have a more severe phenotype [12]. Del Gaudio et al. [16] and Carvalho et al. [15], reported that probands carrying triplications of *MECP2 *show more severe phenotypes and early lethality [15, 16]. In aggregate these studies indicate that i) additional genes mapping to the X chromosome or to autosomes may contribute to the clinical variability, and ii) higher *MECP2* dosage may lead to increased phenotypic severity.

In parallel with the clinical studies, molecular investigation of the genomic rearrangement features in MRXSL families revealed that, similar to the phenotypic heterogeneity, the genomic rearrangement structure is also heterogeneous; individual mutational events exhibit nonrecurrent copy number gains of varying size and gene content [17] ranging from a few hundred kilobases to several megabases [13, 18]. Despite this ‘genomotype variability’, they share a common smallest region of overlap (SRO) measuring 149 kb, which includes the genes *MECP2* and *IRAK1*. Remarkably, complex genomic rearrangements (CGRs), such as inverted triplications and interspersed duplications, are observed to occur in 26–38% [18–20]. In addition to CGRs, translocations and recombinant chromosomes involve copy number variation of additional genes outside of Xq28 [18, 21, 22]. The phenotypic consequences of these various CGRs have not been explored in detail due to small cohort sizes and use of limited genomic studies.

The extensive structural allelic variability at the Xq28 locus may contribute to the clinical variability and phenotypic severity. To explore this hypothesis, we performed detailed clinical and genomic studies on a large cohort (*N* = 137) of molecularly diagnosed probands with MRXSL. Data from genomics, transcriptomics, MECP2 protein studies and human phenotype ontology (HPO) semantic similarity scores were investigated. Genomic results revealed that Tandem duplications occur in approximately half of the cohort. Surprisingly, Terminal duplications are mostly constituted by unbalanced translocations and recombinant chromosomes; these contribute to a large proportion of the de novo events. This results in an ascertainment bias towards larger copy number variants (CNV) sizes and increased number of Xq28 genes with dysregulated gene expression. Quantitative clinical phenotype analysis revealed recognizable rearrangement ‘genomotype’ associated with clinical synopsis of features (i.e., specific patterns); particularly in probands carrying *MECP2* triplication and terminal duplications.

## Methods

### Study population

Subjects were recruited using two main sources: (1) Individuals clinically evaluated at Texas Children’s Hospital Blue Bird Circle Rett Center (TCH-BBC Rett Center, *N* = 82) and (2) subjects from whom biospecimens were submitted for research and clinical information was systematically gathered through local providers or telemedicine (*N* = 55). To increase the robustness of the genomotype-phenotype association study, we included probands with resolved structural variants who were published previously (*N*= 38, Additional file: Table S1 and S2) [15, 16, 23–27]. For patients who were evaluated at TCH-BBC Rett Center, clinical information was obtained by retrospective chart review using H-46044 protocol approved by BCM’s Institutional Review Board (IRB). We used BCM IRB approved H-32407 and H-47281/Pacific Northwest Research Institute WIRB #20202158 protocols to clinically examine patients on a research basis.

For genomic studies, participants were consented according to the IRB at BCM approved protocols: H-29697, H-20268, H-18122, and H-26667 or H-47281/Pacific Northwest Research Institute WIRB #20202158. Whole blood samples (3–10 mL) were collected via peripheral venous blood draw in Ethylenediaminetetraacetic acid (EDTA) and Acid Citrate Dextrose (ACD) vacutainer tubes from probands molecularly diagnosed with MRXSL. Genomic DNA from patients and family members was isolated from blood according to standard procedures.

In total 137 probands (136 males and 1 female, Additional file: Table S1), including 11 affected siblings and one affected uncle carrying Xq28 duplications encompassing *MECP2,* were enrolled for research. Genomic studies included 125 unrelated affected individuals in addition to 112 unaffected mothers who were evaluated for CNV and breakpoint junction carrier status.

### Clinical information

For clinical information, we developed a comprehensive clinical information work log based on our center’s clinical experience and a meticulous literature search to capture the clinical domains and features of MRXSL. We recorded patient’s highest developmental skills for developmental assessment (*e.g.*, if a patient was ambulatory at younger age and then lost walking skills, we specified that the patient achieved ambulation). We included symptoms if they were present at any time point in their life to calculate the most accurate prevalence for given feature (*e.g.*, if a patient had epilepsy in the past and now is seizure-free, we counted epilepsy as present). For missing information from the previously published cases in the work log, we contacted families and/or their local physician to maximize the clinical data gathering.

### Developmental quotients and highest achieved developmental skills

Based on the individual’s reported developmental functioning, a developmental age equivalent was established using the mean age at which specific developmental skills are typically acquired (50th percentile). The developmental quotient (DQ) was then calculated by dividing the individual’s developmental age (DA) by the chronological age (CA) and multiplying by 100 (DQ = DA/CA × 100), giving a DQ ratio. A DQ of < 70–75% indicates delay in the affected area of development [28]. Since an individual’s developmental age is not expected to increase past a given CA (depending on the domain being assessed), DQs are less relevant measurements for older aged individuals. Thus, we also noted the highest achieved gross and fine motor skills in months. Absent to little speech is one of the core features of MRXSL. Thus, we did not calculate speech/language DQ or highest achieved speech skills.

### Genome analysis by custom-designed high-resolution oligonucleotide array Comparative Genomic Hybridization (aCGH)

Probands enrolled in the MRXSL research were initially evaluated for copy number changes in chromosomes X and Y using a custom designed tiling-path oligonucleotide microarray (Additional file: Table S2). This custom 4 × 180 K Agilent Technologies microarray (AMADID #086099, #025384 or #085948) spans the entirety of X and Y with an enhanced coverage around the *MECP2* gene. AMADID #086099 was designed using the Agilent Sure Design Website version 6.9.1.1 (https://earray.chem.agilent.com/suredesign/) on NCBI Build 37. Coordinates and coverage details are described in Grochowski, et al. [29]. Arrays were run according to the manufacturer’s protocol (Agilent Oligonucleotide Array-Based CGH for Genomic DNA Analysis, version 7.2, Agilent Technologies) with modifications (Beck and Carvalho et al., 2019) [30] on samples from probands and parents when available to investigate for the presence of CNVs. Arrays were scanned using Agilent G2600DA SureScan Microarray Scanner System; data were extracted and normalized using Agilent Feature Extraction Software version 12.1.1.1. These data were then imported and analyzed in Agilent Genomic Workbench version 7.0.4.0. Genomic copy number was defined by the normalized log2(Cy5/Cy3) ratio of the CGH signal. The de novo and inherited copy number changes observed in X and Y chromosomes were mapped to GRCh37/hg19 for further genomic analysis.

### Droplet digital PCR

Copy number changes in *MECP2 *and inheritance were confirmed by droplet digital PCR (ddPCR) using the Bio-Rad QX200 system (Hercules, CA) and TaqMan protocol [31]. Custom primer/probe sets in *MECP2* were designed using Primer3Plus Version 2.4.2 (https://primer3plus.com/cgi-bin/dev/primer3plus.cgi) [32] sequence information is displayed in Additional file: Table S3. Primer/probe set is located within exon 3 of *MECP2* transcript NM_001316337.2 (chrX:153,297,701–153,297,851, GRCh37) amplifying 151 bp. The TaqMan probe located within *MECP2* was labeled with the FAM fluorescent tag, and the control primer/probe set containing reference *RPP30* NM_006413.4 (chr10:92,631,746–92,631,819, GRCh37) was labeled with HEX and amplified a 62 bp region. PCR used BioRad ddPCR super mix for probes (No dUTP), 50 ng of template DNA, and a BioRad C1000 Touch Thermocycler, with the following thermocycling conditions: 95 °C, 55 °C, 72 °C with 35 cycles. Samples were run with two replicates. Droplet generation used a BioRad QX200 droplet generator and counted using a BioRad QX200 droplet reader. The individual FAM and HEX signals from each droplet were then quantified and interpreted with the QuantaSoft Analysis Pro Software version 1.0 to make a precise copy number call.

### Genomic optical mapping

Ultra-high molecular weight (UHMW) genomic DNA for use in genomic optical mapping was extracted from blood or cells grown from frozen or fresh lymphoblastic cell lines (LCL) using Bionano Prep™ Blood and Cell Culture DNA Isolation Kit (Bionano Genomics) with an input of 1.5 million cells. Subsequent DNA quantity and size was confirmed using Qubit™ dsDNA BR Assay Kit. A total of 0.75 µg of UHMW DNA was then labeled by DLE-1 using the Bionano Prep direct label and stain (DLS) method (Bionano Genomics) and loaded onto a flow cell to run on the Saphyr optical mapping system (Bionano Genomics). Approximately 500–1800 Gb (150–500 X genome coverage) of data was generated per run. Raw optical mapping molecules in the form of BNX files were run through a preliminary bioinformatic pipeline that filtered out molecules less than 150 kb in size and with less than 9 motifs per molecule to generate a de novo assembly of the genome maps. Data was then aligned to an *in-silico* reference genome (GRCh37, Hg19) using the Bionano Solve v3.7 RefAligner module. Structural variant (SV) calls were generated through comparison of the reference genome using a custom Bionano SV caller. Manual inspection of proposed breakpoint junctions was performed by visualization in the Bionano Access software program v1.7.

### Short-read and long-read whole genome sequencing (WGS)

In total 74 samples from probands underwent short-read genome sequencing (Illumina NovaSeq 6000 platform) (Additional file: Table S2) at the Human Genome sequencing center (HGSC) at BCM on genomic DNA isolated from the whole blood of MRXSL probands and their available family members. All experimental and analysis steps are detailed in Grochowski et al. article [29]. In summary, following sample QC, libraries were prepared with KAPA Hyper reagents and sequenced using the Illumina Novaseq 6000 to generate 150 bp paired-end sequence reads for all samples in a format of multiplexed pools to generate an average of 30X coverage. Post-sequencing data analysis was performed using the HGSC HgV analysis pipeline, which executed base calling, mapping (BWA-mem) to the reference genome (Hg19), merging, variant calling (xAtlas), post-processing, annotation and QC metric collection for all sequencing events. We achieved 96% of the bases covered to a depth of 20X or greater with an average of 37X coverage. A subset of samples (*N*= 22 probands)) had sequencing performed at the National Genomics Infrastructure (NGI), in Stockholm, Sweden using an Illumina 30X PCR-free paired-end (PE) approach [33].

For samples that could not have breakpoint junction established despite aCGH, short-read WGS and optical mapping, we performed long-read genome sequencing using the Oxford Nanopore Technologies (ONT, PromethION, *N* = 15 and MinION *N* = 12) or PacBio HiFi platforms (*N* = 29) (Additional file: Table S2) as recently described in Grochowski et al. [29] and Smolka et al. articles [34]. For both ONT and PacBio platforms, after DNA quality was assessed using Qubit and pulsed-field gel electrophoresis (PFGE), 15 μg genomic DNA was used to construct a library. For ONT, the SQK-LSK110 ligation sequencing kit with an average fragment length of 15 kb. Using the Oxford Nanopore Technologies Promethion instrument, one flowcell was sequenced per library with an average yield of 90 Gb per sample. Basecalling was performed using Guppy version 4.3.4 + ecb2805 and methylation analysis with Megalodon version 2.3.1 [https://github.com/nanoporetech/megalodon] using the default parameters of the program. For PacBio, using the SMRTbell Express Template Preparation Kit 2.0 with an average fragment length of 15 kb. Using the PacBio Sequel IIe instrument, two SMRT cells were sequenced per library for an average of 43 Gb of HiFi reads per sample with an average coverage of 15-20x.

### MinION

In house nanopore sequencing used a MinION R.10.4.1 flow cell, with the V14 ligation sequencing kit (LSK114) following the manufacturer’s directions with modifications. DNA was sheared to a N50 of 10 kb using a g-tube (Covaris), 2 µg of DNA was sheared by centrifugation at 6000 rpm (3381 × g) in an Eppendorf 5424r centrifuge two times for one minute each. Shearing was confirmed by visualization on a 1% agarose gel. DNA ends were repaired using the NEBNext FFPE DNA Repair kit (NEB cat# E7180S) following the manufactures directions. DNA was purified by AMPure magnetic beads. Sequencing adapters ligation was carried out using NEB Quick Ligase (NEB cE7180S) and Oxford Nanopore’s proprietary ligation buffer. Following purification with AMPure magnetic beads. Sequencing runs used approximately 15 fmol of library at a time on a R10.04.1 flow cell (FLO-MIN114). For samples sequenced with region of interest enrichment a bed file with coordinates for the region of interest was prepared for each sample. This region roughly corresponds to the first and last probe from the array ± at least 500 kb (Additional file: Table S4). Adaptive sampling used the most complete reference available, T2T [35]. Real time base calling was used with Guppy 6.0.1 with fast base calling, called bases were mapped to hg19, using minimap2 [36]. When the first adaptive sampling pass did not collect reads spanning the breakpoint, the flow cell was washed with the Oxford Nanopore Flow cell wash kit (cat#EXP-WSH004) and additional library ran until breakpoints were identified.

### Sanger sequencing of breakpoint junctions

Using the CNV coordinates from the customized aCGH, confirmed by the presence of soft-clipped reads at the corresponding CNV coordinates from genome sequencing, we designed primers (Additional file: Table S5) to confirm both junction sequence and inheritance status. PCR was performed using the Q5 Hot Start polymerase (New England BioLabs, NEB# M0494S) or LongAmp Hot Start Taq (NEB# M0533S) annealing temperatures were determined using the NEB Tm calculator (https://tmcalculator.neb.com/#!/main). PCR was conducted on families, along with control primers to ensure that DNA was of sufficient quality for amplification. Confirmation of SV required the presence of the appropriately sized band followed by dideoxy-Sanger sequencing.

PCR products were enzymatically purified using ExoSAP (Thermofisher), in brief, 5 µl of sample was mixed with 2 µL of ExoSAP, incubated at 37 °C for 4 min, followed by 1 min at 80 °C. Following purification, samples were diluted with 14 µL of water, and 1 µl of DMSO. Diluted samples were split and premixed with primers and sent for commercial Sanger sequencing.

Primers to amplify and Sanger sequencing breakpoint junctions are provided in Additional file: Table S5. The genomic approaches are summarized in Additional file: Fig S1.

### RNA Sequencing

#### LCL generation and culture

EBV transfected LCLs were created from the blood collected to ACD tubes in the Tissue Culture Core (TCC) at BCM. In total, *N* = 64 lymphoblast cell lines were generated at the BCM TCC for use in this study. All proband cell lines were generated and banked at the BCM TCC. Lymphoblast cell lines were maintained at ~ 500,000 cells per mL in RPMI 1640 with L-glutamine and 25 mM HEPES (Corning #10–041-CV) + 15% fetal bovine serum + 1% antibiotic/antimycotic (Gibco #15240096). Cultures were maintained at 37 °C in 5% CO_2_ in T25 or T75 flasks. Unaffected control lymphoblast cell lines were purchased from Coriell Institute for Medical Research (labels in Additional file: Table S6) and maintained under the same conditions as the lymphoblast obtained from probands.

#### Fibroblast cell line generation and culture

Three to four mm skin biopsy was obtained and transferred to various medias for fibroblast culture formation at the TCC. In total, *N* = 18 fibroblast cell lines were generated at the BCM TCC for use in this study. All proband cell lines were generated and banked at the BCM TCC. Fibroblast cell lines were maintained at ~ 50% confluency in Dulbecco’s Modified Eagle’s Medium with high glucose (MilliporeSigma #D6429) + 10% fetal bovine serum + 1% antibiotic/antimycotic (Gibco #15240096). Cultures were maintained at 37 °C in 5% CO_2_ in T25 or T75 flasks. Unaffected control fibroblast cell lines were purchased from Coriell Institute for Medical Research (labels in Additional file: Table S6) and maintained under the same conditions as the fibroblasts obtained from probands.

#### RNA isolation

For fibroblast lines, cells were seeded into 6-well plates at a density of 31,250 cells / cm^2^ (300,000 cells per well) and allowed to settle and grow for 48 h. After 48 h, total RNA was isolated using the Qiagen RNeasy Mini kit (Qiagen #74106) according to manufacturer’s protocol including on-column Dnase digestion (Qiagen #79254).

For LCLs, 1 × 10^7^ cells were spun down at 100 rcf for 5 min at room temperature. Media was gently aspirated, and the resulting cell pellet was snap frozen in liquid nitrogen and stored at −80 °C until processing. The cell pellet was rapidly thawed and resuspended in 50 μL PBS + 1 × phosphatase (GenDEPOT #P3200) + 1 × protease inhibitor (GenDEPOT #P3100) + 3μL SUPERase-In RNAse Inhibitor (Invitrogen #AM2696) / mL buffer. 25 μL of cell suspension was added to 350 μL of Qiagen buffer RLT, and RNA was isolated using the Qiagen Rneasy Mini kit (Qiagen #74106) according to manufacturer’s protocol including on-column Dnase digestion (Qiagen #79254).

#### RNA sequencing

RNA was isolated as described above and sent to Genewiz for RNA integrity assessment, library preparation, and sequencing on the Illumina HiSeq platform. For each sample, approximately 30 million 150 bp pair-end reads were generated. Raw reads were trimmed before mapping by Trimmomatic v0.39 using the adapter reference TruSeq3-PE.fa:2:30:10 [37]. Trimmed reads were aligned to GRCh38.p12 version 28 human genome assembly from GENCODE using STAR v2.7.9.a using all default parameters except –sjdbOverhang149 [38].

#### RNA sequencing analysis

Aligned reads were processed using the DESeq2 v1.38.3 analytical package in R [39]. Genes with a median count above 5 were retained for further analysis. The first analysis was to calculate differential gene expression (DEG) between unaffected controls and MRXSL individuals using the design ~ Genotype + Replicate to account for triplicate measurements per line. These DEGs were used to assess global differences in the MRXSL patient samples using principal component analysis. Principal components were calculated using genes with a *p*_adjusted_ < 0.05 and then plotted. Secondary analyses compared individual MRXSL categories using the design ~ Structure, either to unaffected controls or to other categories, as separate contrasts in DESeq2. To assess the distribution of gene expression on an individual gene basis, each triplicate measurement per patient was averaged and then the entire dataset was re-normalized to set the average expression of the unaffected controls to 1. The resulting normalized expression was plotted as a boxplot. These normalized values were used to generate heatmaps of gene expression using the pheatmap package in R. Values in the heatmap were scaled by column and clustered using Euclidean distance.

## Capillary western blot

### Protein isolation

For LCLs, 25 μL of resuspended cells in PBS + inhibitor cocktail (see RNA isolation above) were added to 25 μL of 2 × lysis buffer (4% SDS + 1% NP-40 in 100 mM Tris–HCl pH 7.5 + 1 × phosphatase (GenDEPOT #P3200) + 1 × protease inhibitor (GenDEPOT #P3100) + 2 μL nuclease / mL buffer (Pierce Universal Nuclease for Cell Lysis; ThermoFisher #88702)). Cell lysates were sonicated using a Bioruptor Pico (diagenode #B01060010) using 10 cycles of 30 s sonication and 30 s no sonication, rotated for 20 min at room temperature, and clarified by spinning down at maximum speed for 20 min at room temperature. Clarified lysates were transferred to a fresh tube and stored at −80 °C until further processing.

### Capillary electrophoresis and protein quantification

Protein concentrations were assessed using Pierce BSA assay (ThermoFisher #23275). Lysates were diluted to 0.8 mg/mL in lysis buffer. Samples were prepared for capillary electrophoresis by adding 5 × fluorescence sample buffer (BioTechne #PS-ST01EZ-8) for a final concentration of 1 × sample buffer and boiled for 5 min. Samples were loaded onto a 25-capillary 12–250 kDa fluorescence separation module (BioTechne #SM-FL004) according to manufacturer’s default protocol in batches; each batch contained 5–6 unaffected controls. Total protein stain was used for normalization (BioTechne #AM-PN01). Primary antibody solution of anti-MECP2 (rabbit monoclonal D4F3, Cell Signaling Technology #3456, RRID:AB_2143849; 1:50 dilution). Secondary antibody cocktail of anti-Rabbit HRP (Rabbit detection module; BioTechne #DM-001) was used and exposed per manufacturer’s default recommendation. The Compass software was used to quantify MECP2 signal intensity and total protein intensity using Gaussian curve fit. MECP2 signal was normalized to the total protein signal and each batch was internally normalized to the average normalized MECP2 intensity of the unaffected controls.

### HPO analysis

#### Grouping/core phenotype

Phenotypic characteristics were annotated with Human Phenotype Ontology (HPO) terms for each of the affected individuals (*N* = 136). Five individuals were excluded from analysis due to either too few or too many HPO terms, resulting in 131 affected individuals in the final analysis. The 131 probands were sorted into 5 distinct groups based on their genomic structure as described below in the Genomic Information subsection of Results. The *MECP2* Triplication (TRP) group (*N* = 5) included individuals with triplication of the entire *MECP2*. The “Terminal DUP translocation” group (*N* = 16) consisted of individuals with Xq terminal duplication and translocation. The “Terminal DUP” group (*N* = 10) encompassed individuals with terminal duplication without translocation. The largest group (*N* = 63) comprised of individuals with “Tandem duplications”, while those with structural variations noncompatible with other groups were categorized under "Other complex” (*N* = 37). Each group was assigned a clinical synopsis of core phenotype/HPO terms determined by the frequency of HPO terms within group. An HPO term was considered a core feature if its frequency within a group was above 50%.

#### Similarity matrix

From the HPO resource page (https://hpo.jax.org/app/), data were collected pertaining to OMIM known disease genes (*N* = 42), located on the *p* (hg19 chrX: 0–1,731,000) and *q *arms (chrX:138,733,000–155,270,560) of chromosome X, and their associated HPO terms. These genomic ranges were chosen to encompass all terminal duplications and regions affected by CNVs spanning chromosome X in this cohort. Utilizing OntologyX R packages [40], individual similarity matrices between core phenotypes for the 5 distinct *MECP2 *proband groups and OMIM known disease genes on chromosome X were computed using Lin's semantic similarity score alongside the average method [41–43]. These matrices were then utilized to generate distance matrices for further assessing phenotypic similarity. Hierarchical agglomerative clustering (HAC) employing Ward's method [44] was used on distance matrices to separate them into phenotypically similar clusters, with the determination of the number of clusters being guided by the visualization of the gap statistic curve. The gap statistic was calculated for cluster numbers ranging from k = 1 to 20, and the slope of the gap statistic curve was utilized to determine the optimal number of clusters. Visualization of phenotypic similarity scores across distinct *MECP2 *proband groups and OMIM known disease genes on chromosome X was conducted using the ComplexHeatmap package in R [45]. Subsequently, an analysis was performed examining groups in relation to genes using HPO annotations.

#### Frequency plot

The frequency of each phenotype within each of the distinct *MECP2 *proband groups was calculated. Visualization of the phenotypic frequency in each group was carried out using the ComplexHeatmap package in the R language [45].

#### Statistical analysis

We used both descriptive and inferential statistical analysis. We used the occurrence of each specific clinical feature (*i.e.*, the percentage of MRXSL patients with that feature), using means and standard deviation (SD) for continuous variables, or raw numbers and percentages of the population for categorical and ordinal variables.

Pearson Chi-Square tests were used to find differences between the categorical variables. For the values which reached to statistical significance, we did multiple comparisons to investigate the differences between genetic subgroup using Python programming language (Spyder IDE, https://www.spyder-ide.org). We used Spearman’s correlation to explore the relationships between genetic subgroup and each clinical domain.

We performed one-way ANOVA test to assess continuous variables in different genetic subgroups (*N* = 5). For the statistically significant variables, we conducted Post-hoc analyses including Tukey's test (if variance were homogeneous) and Tamhane’s T2 test (if variance were non-homogeneous) for multiple comparisons. Kruskal–Wallis tests were used to detect differences in the mean scores of clinical feature and different genomic subgroups.

We used Kaplan Meier Survival analysis including pairwise comparison with Breslow (generalized Wilcoxon) test to evaluate survival duration. We investigated the impact of genetic variants on the survival using Cox-regression method by odds ratio. MedCalc® Statistical Software version 22.009 was used to draw survival curve plot.

Statistical analyses used for RNA-sequencing are described above. Both Spearman and Pearson correlation were calculated between the paired log-transformed *MECP2* RNA and MECP2 protein per LCL sample.

A* p*-value < 0.05 was considered to be statistically significant. We used IBM SPSS Statistics for Windows, Version 29.0. Armonk, NY: IBM Corp for all statistical analyses.

## Results

### Study subjects

A total of 137 individuals from 125 families were enrolled (Additional file: Table S1). Age ranged from 3 weeks old (BAB3053, who died at 3 weeks and carried a triplication including *MECP2*) to 53 years old (BAB14550, who carried other complex duplication). We were able to obtain updated clinical information from 18 out of 38 published individuals. Eighty-two subjects were clinically evaluated at TCH-BBC Rett Center. The remaining 55 subjects’ clinical information were gathered through a mixture of telemedicine and local provider information. One hundred thirty-six of the subjects were male and only one female was included since she was an unbalanced translocation carrier between chromosome 13 and X, thus clinical presentation was classical MRXSL phenotype.

### Genomic rearrangement studies

Samples from all probands were submitted to a customized aCGH to confirm the clinical and molecular diagnosis of *MECP2* duplication. After confirmation, short-read sequencing was performed in all unpublished, newly enrolled probands and for previously published samples that did not have their breakpoint junction resolved by Sanger sequencing (*N*= 74) [15, 18, 20]. Long-read genome sequencing (*N* = 42) and Optical Genome Mapping (OGM, *N* = 53) were performed on all samples displaying aCGH profile suggesting a complex rearrangement involving the *MECP2* locus (*i.e.*, interspersed duplications or triplications flanked by duplications) as well as those with an apparently simple duplication for whom short-reads did not resolve breakpoint junctions. The workflow of the genomic approaches is shown in Additional file: Fig S1. In all, combined analysis of customized aCGH, short-read and long-read genome sequencing and OGM allowed us to resolve the genomic structure of 118 (94%) out of 125 unique *MECP2* duplications. Of the seven unsolved structures, two are terminal duplications for whom none of the methodologies applied could resolve, four only had biological material for aCGH, one had aCGH and short-read sequencing without resolving the genomic structure. Additional file: Table S2 details the genomic platform utilized for each sample in this cohort and Additional file: Table S7 provides the detailed coordinates of identified CNVs.

**Fig. 1:**
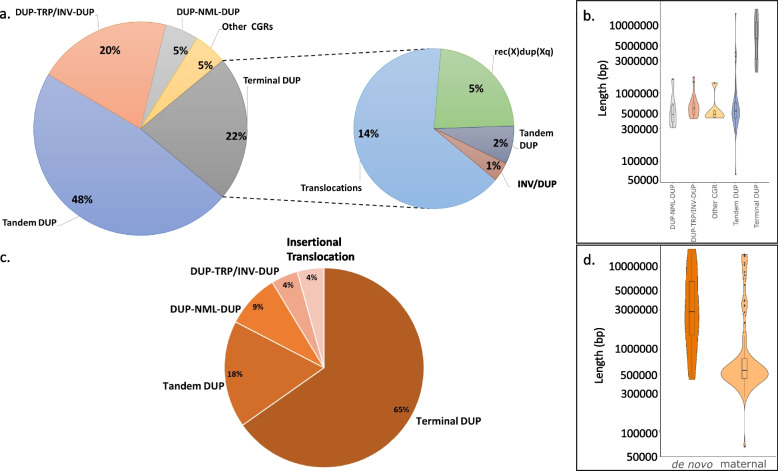
Distribution of genomic structures observed in the MRXSL cohort. **a** Pie chart distribution of the genomic structure of 118 unrelated individuals carrying *MECP2* duplication. **b** Violin plot representing the size distribution of the CNV encompassing *MECP2* in each genomic subgroup. Length in bp, log10 scale. **c** Pie chart distribution of the genomic structure of 23 de novo* MECP2* duplication events. **d** Violin plot representing the size distribution of inherited and de novo structural variants in MRXSL. CGR: Complex Genomic Rearrangement, DUP: Duplication, INV: Inverted, NML: Normal, rec: Recombinant, SV: Structural Variant, TRP: Triplication

The resolved genomic structure of the Xq28 duplication spanning *MECP2* encompasses the following five categories or rearrangement types: head-to-tail duplication (tandem duplication, 48%), inverted triplication flanked by duplications (DUP-TRP/INV-DUP, 20%) [15], terminal duplications (*i.e.*, duplications including the pseudoautosomal region 2- PAR2, 22%), interspersed duplications (DUP-NML-DUP, 5%) and other types of complex duplication rearrangements (5%) (Fig. 1, Additional file: Fig S2 and Additional file: Table S7). Most of the terminal duplication structures consist of translocations (65%), followed by recombinant chromosomes (23%), large tandem duplications (8%) and inverted duplication (4%). Of the 17 subjects with translocation, 10 of them involve chromosome Y (59%). Triplication encompassing *MECP2* were observed in two groups, four individuals carry DUP-TRP/INV-DUP while one carries a terminal duplication with translocation to Yq.

Duplication size varied from 64.6 kb (BAB12190, partial *MECP2* duplication) to 16.5 Mb (BAB3212) with hg19 genomic coordinates spanning from chrX:138,733,695 to the telomere. Median size of the *MECP2* duplications varies from 484 to 541 kb in all groups with at least 75% < 700 kb except for Terminal duplications in which median is 6.2 Mb with 75% of the duplications < 10.5 Mb (Fig. 1b). CNV inheritance was investigated by ddPCR using probes targeting *MECP2* as well as by checking the presence of breakpoint junctions of the SV. The latter was used to rule out balanced events in the maternal X chromosome either by breakpoint junction Sanger PCR or by short-read sequencing when available. Maternal samples were available for 110 families indicating that 87 out of 110 (79%) CNVs were inherited from an apparently unaffected mother, whereas 23 events occurred de novo in the proband (Additional files: Table S1/S2 and Fig. 1d). Notably, 15 out of 23 (65%) de novo events occurred in the Terminal duplication group, 13 out of 15 (87%) consisting of translocations which reveals a strong bias towards that specific genomic structure to the de novo events in MRXSL. Only 17% of probands with Tandem duplication carry a de novo event, whereas complex rearrangements such as DUP-NML-DUP (8%) and DUP-TRP/INV-DUP (4%) are rarely de novo (Fig. 1c). Importantly, de novo events of MRXSL duplications tend to be larger than inherited CNVs with mean size of 2.9 Mb compared to 550 kb (Fig. 1d).

Genomic content in MRXSL varies largely and partially depends on whether the structure is a terminal duplication or any other SV. In all, the largest duplication events will include 40 genes with disease association in OMIM, a few of them known to affect the nervous system when disrupted or dysregulated including *FMR1* (*309550), *L1CAM* (*308840), *FLNA* (*300017), *GDI* (*300104), *RAB39B* (*300774) (Additional file: Fig S2a). The SRO in our cohort of probands with a clinical diagnosis consistent with MRXSL (*N* = 136 probands; BAB12190 was excluded since he was healthy due to partial *MECP2* Duplication) is 127 kb [chrX:153,259,853 (BAB3039)−153,386,785 (BAB15790)]. Importantly, this SRO includes both *MECP2* and *IRAK1* but also the majority of the *MECP2* noncoding cis-regulatory elements (CREs) identified by interspecies sequence comparison and reporter plasmid transfection assays [46] or more recently *Mecp2* mouse brain CRE that are conserved in humans [47] (Additional file: Fig S2b).

### Clinical characteristics of MRXSL

We used the genomic structure information as a general guide to perform the genotype–phenotype analysis: Tandem duplication, Other complex duplications (this group includes genomic rearrangements that do not fit into the remaining subgroups such as DUP-TRP/INV-DUP, DUP-NML-DUP and other CGRs), Terminal duplications, Translocations and Triplications including *MECP2*. Of note, the Translocation subgroup includes translocations to other chromosomes but not insertional translocations (*N* = 1). All translocations in this subgroup also had terminal duplication, thus Translocations are a subgroup of Terminal duplication. Previous studies have shown that individuals carrying triplication encompassing the entire *MECP2 *coding region present with a more severe phenotype [15, 16], therefore, we analyzed *MECP2* triplications all together regardless of the genomic structure. It has also been suggested that individuals carrying duplication of *RAB39B *present a more severe phenotype as well as translocations [12, 13]. To independently investigate the potential contribution of *RAB39B* and translocation to phenotypic severity we separated patients carrying terminal duplication from those with terminal duplication with translocations. With the purpose of analyzing the clinical data, we have included seven individuals with unsolved genomic structures to the groups as following: apparently simple duplications were included in the Tandem duplication group and terminal duplication/unsolved structure were included in the Terminal duplication group.

### Pre-/peri-/post-natal

Birth weight was available in 100 subjects, 83 had normal birth weight, 11 subjects were small for gestational age (SGA) and six subjects were large for gestational age (LGA). The rate of normal birth weight gradually decreased in the following order Tandem duplication (92.1%), Other complex duplication (88.2%), Terminal duplication (66.6%), Translocation (71.4%) and Triplication (40.0%). Statistically significant correlation was observed between groups (*p*-value = 0.014, Table 1). The main difference was between Tandem duplication and Triplication, and Other complex duplication and Triplication (Additional file: Table S8).

**Table 1:** Clinical features with frequency (%) for each genetic subcategory and statistical difference between groups (last column)

	**Tandem Dup**	**Other Complex Dup**	**Terminal Dup**	**Translocation**	**Triplication**	***p*-value**
**C-section delivery**	19/40=47.5%	13/31=41.9%	3/8=37.5%	7/11=63.6%	4/5=80.0%	0.415
**Full-term delivery**	57/63=90.4%	28/37=75.6%	8/11=72.7%	11/13=84.6%	3/5=60.0%	0.158
**Normal birth weight**	35/38=92.1%	30/34=88.2%	6/9=66.6%	10/14=71.4%	2/5=40.0%	**0.014**
**Postnatal complications**	21/57=36.8%	19/35=54.3%	9/11=81.8%	13/16=81.3%	4/4=100%	**0.001**
**Congenital Hypotonia**	30/59=50.8%	16/31=51.6%	9/11=81.8%	14/15=93.3%	5/5=100%	**0.004**
**Pneumonia**	49/66=74.2%	25/37=67.5%	9/11=81.8%	13/17=76.4%	3/4=75%	0.886
**Recurrent URI**	46/56=82.1%	24/33=72.7%	6/9=66.6%	11/15=73.3%	3/4=75%	0.766
**Urinary Tract Infections**	11/48=22.9	9/29=31.0%	5/8=62.5%	9/13=69.2%	0/1=0%	**0.01**
**Microcephaly**	2/53=3.7%	2/23=8.6%	5/9=55.5%	10/13=76.9%	2/3=66.6%	**0.001**
**Normal weight**	48/57=84.2%	20/25=80.0%	7/9=77.7%	12/14=85.7%	1/1=100%	0.955
**Normal height**	48/58=82.7%	19/24=79.1%	4/8=50.0%	6/11=54.5%	1/1=100%	0.102
**Dysmorphism**	38/47=80.8%	19/21=90.4%	8/9=88.8%	13/13=100%	5/5=100%	0.329
**Chewing/swallowing difficulty**	55/60=91.6%	32/36=88.8%	9/11=81.8%	14/16=87.5%	5/5=100%	0.792
**Tube feeding**	21/60=35.0%	12/32=37.5%	6/9=66.6%	8/14=57.1%	5/5=100%	**0.019**
**Constipation**	56/60=93.3%	31/35=88.5%	8/11=72.7%	16/16=100%	4/5=80.0%	**0.13**
**GERD**	48/60=80.0%	28/36=77.7%	8/11=72.7%	14/16=87.5%	4/5=80.0%	0.907
**CAKUT**	17/59=28.8%	15/36=41.7%	8/11=72.7%	12/15=80.0%	5/5=100%	**0.001**
**Abnormal tone**	53/57=92.8%	31/32=96.8%	8/10=80.0%	14/15=93.3%	2/2=100%	0.473
**Epilepsy**	39/66=59.0%	18/36=50.0%	5/11=45.4%	10/17=58.8%	2/5=40.0%	0.779
**Movement anomaly**	25/46=54.3%	14/32=43.7%	1/8=12.5%	3/11=27.2%	3/3=100%	**0.04**
**Neurobehavioral disorder**	55/64=85.9%	32/36=88.8%	10/11=90.9%	12/16=75.0%		0.561
**Dysautonomia**	54/64=84.3%	33/35=94.2%	8/10=80.0%	10/12=83.3%		0.464
**Bruxism**	38/56=67.8%	26/33=78.7%	5/9=55.5%	12/14=85.7%		0.288
**High pain tolerance**	41/53=77.3%	25/33=75.7%	7/9=77.7%	12/14=85.7%		0.898
**Musculoskeletal problems**	25/43=58.1%	16/29=55.1%	2/7=28.5%	4/12=33.3%	2/2=100%	0.213
**Insomnia**	34/60=56.6%	17/35=48.5%	3/9=33.3%	8/14=57.1%		0.556
**Sleep apnea**	27/56=48.2%	16/32=50.0%	6/9=66.6%	12/15=80.0%	2/2=100%	0.121
**Hearing deficit**	3/54=5.5%	4/31=12.9%	1/10=10.0%	3/15=20.0%	3/3=100%	**0.001**
**Vision problems**	31/53=58.4%	17/33=51.5%	9/11=81.8%	10/16=62.5%	4/4=100%	0.203
**Imaging abnormality**	26/31=83.8%	6/10=60.0%	5/7=71.4%	9/9=100%	4/4=100%	0.151

*C-section* Caesarian section, *CAKUT* Congenital Anomalies of the Kidney and Urinary Tract, *Dup* Duplication, *GERD* Gastroesophageal Reflux Disease, *URI* Upper Respiratory Infection. Statistically significant values are bolded

Birth height measurements were available in 58 subjects. Fifty-two of them had normal length, four subjects were shorter, and two subjects measured longer for gestational age. Head circumference was available in only 29 subjects, 27 subjects were normocephalic, and there was one microcephalic and one macrocephalic subject.

Postnatal complications requiring extended hospital stay including NICU admission, were observed in 66 out of 123 (53.6%) subjects and this ratio increased in the order Tandem duplication (21/57 = 36.8%), Other complex duplication (19/35 = 54.3%), Terminal duplication (9/11 = 81.8%), Translocation (13/16 = 81.3%) and Triplication (4/4 = 100%). There was a strong statistical correlation between groups (*p*-value = 0.001, Table 1). The statistical differences were observed between Tandem duplication and Terminal duplication, Translocation and Triplication (Additional file: Table S8).

One of the defining features of MRXSL is congenital hypotonia (considered cut off as 4 months or younger) and it is observed in 74 out of 121 (61.1%) of subjects, and the rate increased in the order of Tandem duplication (30/59 = 50.8%), Other complex duplication (16/31 = 51.6%), Terminal duplication (9/11 = 81.8%), Translocation (14/15 = 93.3%) and Triplication (5/5 = 100%). There was a strong statistical correlation between groups (*p*-value = 0.004, Table 1) and this significance was mainly due to difference between Tandem duplication/Other complex duplication and Translocation (Additional file: Table S8).

### Developmental parameters

**Fig. 2:**
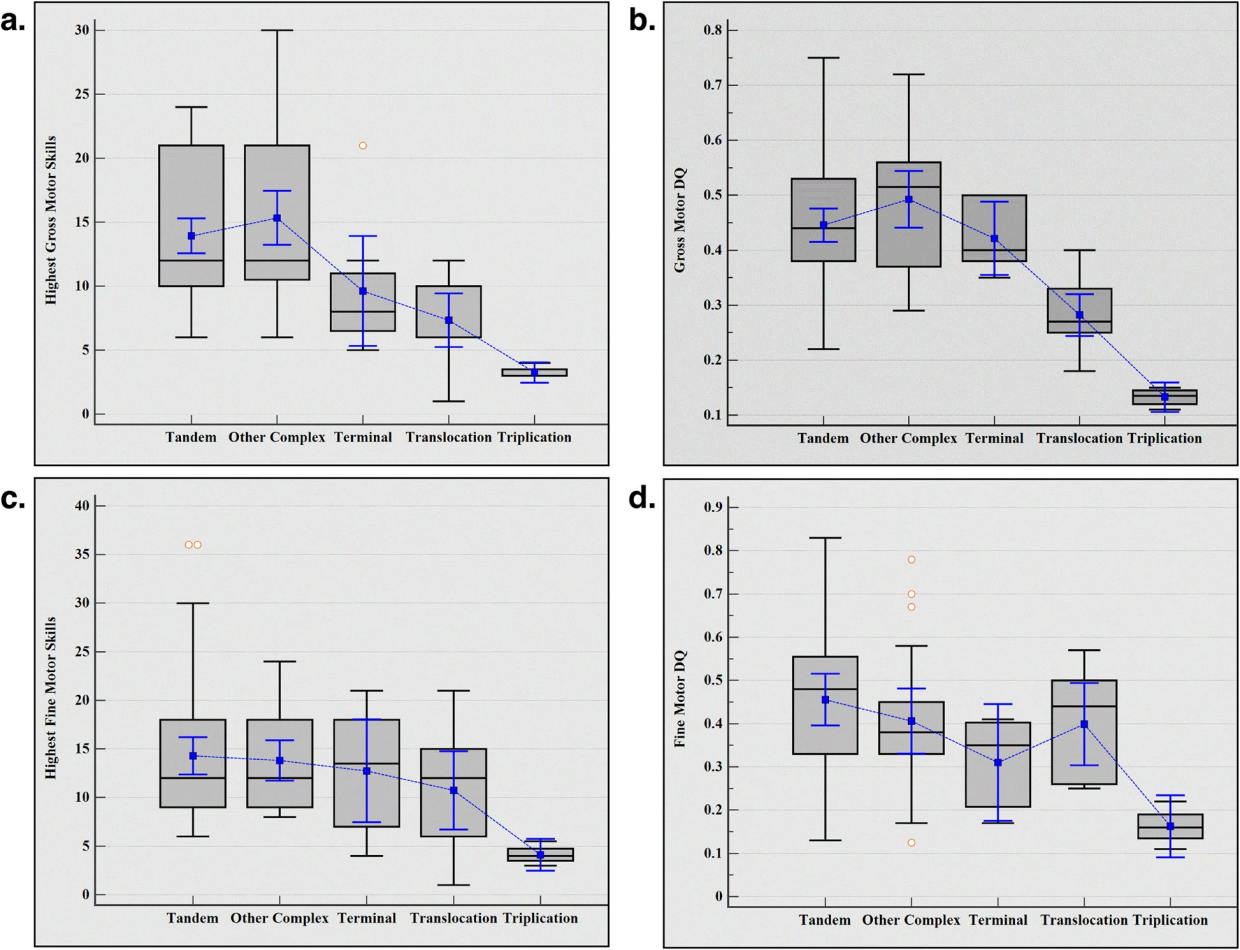
Developmental Comparisons of Different MRXSL structural variants. Highest achieved gross motor skills (**a**), gross motor DQ (**b**), highest achieved fine motor skills (**c**), fine motor DQ (**d**). Overall, the developmental delay severity worsens in the following order: Tandem duplication < Other complex duplication < Terminal duplication < Translocation < Triplication. Medians are provided in gray boxes/black lines, means are given in blue lines. MRXSL: *MECP2* Duplication Syndrome, DQ: Developmental Quotient

All patients have severe to profound DD/ID with highest developmental skills mostly not exceeding 24 months in all three domains; calculated gross and fine motor skills are provided in Additional file: Table S1. Highest achieved developmental skills, and fine and gross motor DQs gradually declined from Tandem duplication to Triplication (Fig. 2). As a reflection of this, while two of the Terminal duplication individuals achieved independent walking, none of the Translocation and Triplication subjects achieved this important milestone.

Subject BAB12190 carries a ~ 64 kb partial duplication of *MECP2* including the first two exons who presented with normal development and unremarkable physical examination.

### Recurrent infections

One of the core clinical features of MRXSL is the frequent infections [6, 22] which are often reported as recurrent respiratory infections. We evaluated recurrent infections in four categories including pneumonia (PNA), upper respiratory infections (URI), urinary tract infection and others. Pneumonia and upper respiratory infections were frequent and ranged between 67–82% of groups with no statistical difference between groups (Table 1). Urinary tract infections (UTIs) were identified in 34 out of 99 (34.3%) of individuals. The frequency of UTIs gradually increased in the following order Tandem duplication (11/48 = 22.9%), Other complex duplication (9/29 = 31.0%), Terminal duplication (5/8 = 62.5%) and Translocation (9/13 = 69.2%). There is not enough information for individuals carrying Triplication except for one who did not have UTI. Difference between groups was statistically meaningful (*p*-value = 0.01, Table 1); it mainly stemmed from the difference between Tandem duplication and Translocation (Additional file: Table S8). Also, additional infections including acute otitis media (*N* = 42), recurrent skin infections (*N* = 8), sepsis (*N* = 7), and meningitis (*N* = 3) along with low immunoglobulins (*N* = 11) were reported. Only 17 individuals had undergone some immune workup and 15 of them had some abnormalities. Details of these workups are included in the Column S of Additional file: Table S1. The most common abnormality was low IgA level and it is found in eight individuals. Two individuals had poor antibody formation response to vaccines. The remaining immune abnormalities differed and were unique to individual patients.

### Anthropometric measurements

Weight, height and head circumference (OFC) were gathered from subjects’ clinical notes. Percentiles scattered throughout the growth chart, but majority had normal weight (88 out of 106 subjects = 83.0%), height (78 out of 102 subjects = 76.4%) and OFC (62 out of 101 subjects = 61.3%) measurements. Underweight and overweight were observed in 11 (10.3%) and seven (6.6%) subjects, respectively. Short stature was observed in 23 (22.5%) subjects whereas, tall stature was seen in only two (1.9%) subjects. Microcephaly and macrocephaly were observed in 21 (20.7%) and 18 (17.8%) of subjects, respectively. The rate of microcephaly progressively increased from Tandem duplication to Triplication group: Tandem duplication (2/53 = 3.7%), Other complex duplication (2/23 = 8.6%), Terminal duplication (5/9 = 55.5%), Translocation (10/13 = 76.9%) and Triplication (2/3 = 66.6%). There was a strong statistical correlation between groups (*p*-value = 0.001, Table 1). These differences can be attributed to many differences between groups including Tandem duplication-Terminal duplication, Tandem duplication-Translocation, Tandem duplication-Triplication, Other complex duplication-Terminal duplication, Other complex duplication-Translocation and Other complex duplication-Triplication.

### Dysmorphia

Dysmorphic features were reported in all groups and highly common (> 80%) without significant difference between groups (Table 1). There was no distinctive facial gestalt, recognizable dysmorphic features; however, the most common dysmorphic features include facial hypotonia/open mouth, brachycephaly, plagiocephaly, high forehead/frontal bossing, midface hypoplasia/flat nasal bridge, dysplastic ear including large ear, hypo- or hyper-telorism and tapering fingers.

### Gastrointestinal system

We split gastrointestinal (GI) system problems into four domains including: chewing/swallowing difficulties, gastroesophageal reflux, constipation/diarrhea, and other GI issues. For chewing/swallowing difficulties, we attempted to gather information on the severity of chewing/swallowing difficulty by obtaining history on whether patients can eat regular/soft/chopped diet and G-tube dependence status. Feeding and chewing difficulties were highly prevalent in MRXSL (over 80% in all groups without statistical difference, Table 1); however, requirement for a tube feeding increased from Tandem duplication to Triplication group: Tandem duplication (21/60 = 35.0%), Other complex duplication (12/32 = 37.5%), Terminal duplication (6/9 = 66.6%), Translocation (8/14 = 57.1%) and triplication (5/5, 100%). The correlation between groups was statistically significant (*p*-value = 0.019, Table 1). This difference was mainly due to Tandem duplication/Other complex duplication and Triplication (Additional file: Table S8).

Constipation was highly prevalent in all groups with > 80% frequency. Similarly, gastroesophageal reflux disorder (GERD) was reported in > 70% of individuals with MRXSL in all groups and there was no statistically significant difference between groups (Table 1). Of note, intestinal pseudo-obstruction was reported in two out of three Triplication individuals.

### Genitourinary system

Genitourinary (GU) system abnormalities were investigated in two categories including structural [*i.e.*, cryptorchidism, Congenital Anomalies of the Kidney and Urinary Tract (CAKUT)] and functional GU anomalies (*e.g.*, urinary retention and kidney stone). One-hundred and twenty-six subjects had information on GU abnormalities. Anatomical GU defects were reported in 45.2% of individuals (57/126). Importantly, the frequency and complexity of GU defects increased with genomic complexity: Tandem duplication (17/59 = 28.8%), Other complex duplication (15/36 = 41.7%), Terminal duplication (8/11 = 72.7%), Translocation (12/15 = 80.0%) and Triplication (5/5 = 100%). There was a strong statistical correlation between groups (*p*-value = 0.001, Table 1). This significance was due to multiple difference between groups including Tandem duplication and Terminal duplication/Translocation/Triplication, and Other complex duplication and Translocation/Triplication (Additional file: Table S8). Majority of Tandem duplications had relatively minor GU anomalies including cryptorchidism (*N*:8/17 = 47.0%), while hypospadias, hydrocele and hypogenitalia/microphallus were observed in three individuals (17.6%). On the other hand, Terminal duplication, and Translocation and Triplication patients presented with hypogenitalia/microphallus more often (3/8 = 37.5%, 3/12 = 25% and 2/5 = 40%, respectively). Additionally, urinary retention and kidney stone were observed in 16 and 7 subjects, respectively. Similarly, urinary retention and kidney stone were more common in the Tandem duplication group (*N* = 7/59 and 4/59, respectively), while 3/5 of the Triplication subjects had hydronephrosis/vesicoureteral reflux.

### Neurological system

Neurological evaluation was performed in eight categories including tone, epilepsy, movement disorders, behavioral problems, dysautonomia, bruxism, sensory abnormalities/high pain tolerance and others.

- Tone: Abnormal tone such as hypotonia, hypertonia and central hypotonia-appendicular hypertonia was widely prevalent across all groups ranging from 80 to 100%. There was no statistical difference between groups (Table 1).
- Epilepsy: The frequencies of epilepsy in Tandem duplication, Other complex duplication, Terminal duplication, Translocation duplication and Triplication were 59.0%, 50.0%, 45.4%, 58.8% and 40.0%, respectively. Difference between groups was not statistically significant (Table 1). We then investigated the age of seizure onset in these groups and identified that seizures start earlier as the complexity increases. Age of onset for seizure in Tandem duplication, Other complex duplication, Terminal duplication, Translocation duplication and Triplication are 8.4 years, 8.2 years, 5.6 years, 4 years 10 months, and < 2 years, respectively.

We also investigated the presence of neuromotor regression and whether there was a link between regression and epilepsy. Regression was attributed to seizure onset in 12 individuals, seizures becoming refractory in 17 individuals, infections in 6 individuals, and antiseizure medication side effect in 4 individuals.

iii) Movement disorders: There are no MRXSL-specific recognizable movement anomalies. We observed a broad range of abnormal movements including dystonia, ataxia, choreiform movements and spasticity. The frequency varied significantly. One out of eight (12.5%) individuals of Terminal duplication had ataxia, while three out of three (100%) individuals with Triplication had spasticity. There was borderline statistical significance between groups with *p*-value of 0.04 without a specific difference between subgroups (Table 1 and Additional file: Table S8).
iv) Neurobehavioral disorders: Neurobehavioral traits particularly autism spectrum disorder (ASD) was shown to be prevalent in MRXSL [24, 26]. We investigated the neurobehavioral traits in our large cohort. We did not deeply investigate whether patients fulfill ASD criteria however we documented whether there were repetitive movements, poor eye contact and sensitivity to stimulation (all patients have lack of or poor speech thus we queried other features of ASD). One hundred and nine individuals out of 127 (85.8%) had additional at least one of the three core features on top of poor speech. There was no difference between genetic subgroups (Table 1). Of note, individuals with Triplication were mostly too young to assess the neurobehavioral trait. In addition to ASD phenotype, there was only one individual with attention deficit hyperactivity disorder in the entire cohort.
v) Dysautonomia/Bruxism/High Pain Tolerance/Self-mutilation: Dysautonomia features including drooling, blood-flow dysregulation to extremities and abnormal breathing (breath-holding or hyperventilation) are commonly reported in the allelic Rett syndrome and MRXSL [13, 48]. We identified the frequency of dysautonomia in 105/121 (86.7%) of individuals. Bruxism was reported in 81/112 (72.3%) of subjects. High pain tolerance was present in 85/109 (77.9%) of MRXSL individuals. There was no statistical difference between groups for dysautonomia, bruxism and high pain tolerance (Table 1). While 18 individuals reported self-mutilation, 16 subjects stated no self-mutilation.

### Musculoskeletal system

Musculoskeletal anomalies are common in neurodevelopmental disorders due to deconditioning, immobility and nutritional deficiency. We obtained data on 93 subjects for their musculoskeletal problems and 49 (52.6%) reported musculoskeletal abnormalities, with the most common ones including bone fractures (26 subjects), osteopenia/osteoporosis (13 subjects including 3 requiring alendronate infusion), scoliosis (13 subjects), joint contractures (nine subjects). Also, one individual had osteosarcoma. Difference between groups were not statistically significant (Table 1).

### Sleep disorders

Sleep problems in MRXSL were reported in two studies [9, 13]. We evaluated sleep disorders in two categories: insomnia and sleep apnea. We had information on insomnia from 118 subjects and 62 of them (52.5%) were found to have insomnia. Data on sleep apnea status were available in 114 subjects and 63 subjects (55.2%) were reported to have sleep apnea. Only two subjects were reported to have central sleep apnea and the remaining had obstructive sleep apnea. The frequency of sleep apnea increased in the following order: Tandem duplication 27/56 = 48.2%, Other complex duplication 16/32 = 50.0%, Terminal duplication 6/9 = 66.6%, Translocation duplication 12/15 = 80.0% and Triplication 2/2 = 100%. Despite the gradual increase in percentage with worsening complexity, there was no statistical correlation (Table 1).

### Vision and hearing

Ascertainment of vision and hearing abnormalities have been limited in MRXSL. Miguet et al. studied presence of hypermetropia and hearing loss without further details. [7] Information on visual abnormalities were present in 117 subjects and 71 of them (60.6%) reported various, relatively minor visual abnormalities including refraction errors and strabismus. Differences between groups were not statistically significant (Table 1). Individuals who carry Triplication had more serious visual problems including one individual with nystagmus and large cornea, a second individual with nystagmus and hazy cornea, and a third individual with optic nerve hypoplasia. Hearing abnormalities are found to be less common.

Hearing evaluation data were gathered on 113 subjects and only 14 subjects (12.3%) were found to have hearing deficit including two of them with sensorineural hearing deficit. Similar to eye findings, all three Triplication subjects who had information on hearing had hearing loss (100%) and one of them was documented as sensorineural hearing deficit. Thus, a statistical difference was found between groups (*p* = 0.001, Table 1). The difference between groups were due to Triplication and all other subgroups (Additional file: Table S8).

### Neuroimaging findings

Neuroimaging abnormalities were reported in MRXSL previously. Brain MRI or CT results from 61 subjects were available. In 50 of them, imaging studies revealed various non-specific abnormalities, with the most common including corpus callosum hypoplasia, delayed myelination, cerebral atrophy and ventriculomegaly. Of note, all Translocation and Triplication subjects had abnormalities. There was no statistical correlation between groups (Table 1).

### Other clinical findings

We also investigated other problems not commonly reported in MRXSL. Eczema, asthma, anemia (mostly iron deficiency anemia), milk protein allergy were the most common additional findings. Of note, four subjects had hyponatremia, three subjects had bleeding diathesis or thrombocytopenia, four subjects had various short stature disorders including Leri-Weill Dyschondrosteosis (BAB2684), rhizomelic shortening (BAB3037) and dwarfism (BAB3212 and BAB14298). BAB2684, BAB3037 and BAB14298 probands carry Terminal duplications resulting from a recombinant X chromosome therefore short statures are likely related to the deletion of *SHOX*. The fourth individual with short stature (BAB3212) does not have Xp (*SHOX*) deletion but a 3q29 translocation. Patient was clinically diagnosed as atypical dwarfism due to growth hormone deficiency. Thus, the short stature in this individual has a different etiology. Lastly, three subjects had various autoimmune disorders including relapsing polychondritis (BAB2806), recurrent pleural/pericardial effusions (BAB14598) and ichthyosis (BAB3037).

### Survival analysis reveals evidence for influence of genomotype

**Fig. 3:**
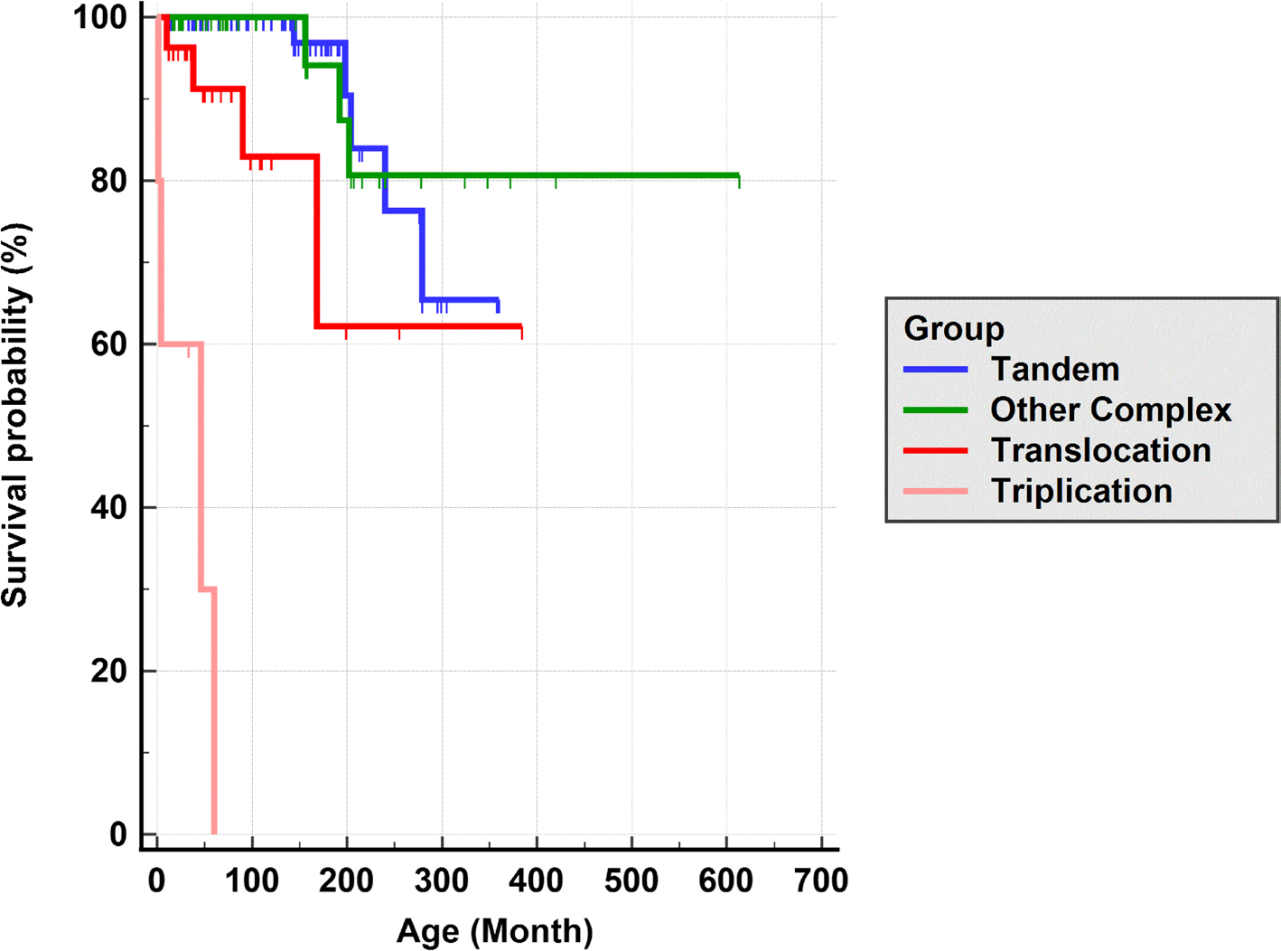
Survival curve analysis for different genomic subgroups. Cox regression for survival analysis indicate that SVs are good predictor of survival probability: Tandem (5/59) > Other complex (3/41) > Translocations (4/16) > Triplications (4/5) (Additional file: Table S1). Overall *p*-value < 0.0001. SV: Structural Variant

Since all Triplication subjects either died or were gravely ill within the first few years of life, we investigated whether different genomic subgroups play a role in the survival of individuals with MRXSL. Kaplan Meier/Cox Regression survival analysis showed that survival duration gradually decreases from Tandem duplication to Triplication (Fig. 3). The difference between groups were due to Triplication and all other subgroups, and Tandem duplication and Translocation (Additional file: Table S8). Cox regression for survival analysis indicated that different structural variations are statistically significant predictor of survival duration (*p*-value < 0.001). Strikingly, the likelihood of death compared to Tandem duplication had increased 147 times (95% Confidence Interval, 22.30–978.37) in the Triplication group and 4.26 times (95% Confidence Interval, 1.13–16.12) in the Translocation group. Of note, none of the Terminal duplication (*N* = 11) individuals died. However, six out of 11 of the Terminal duplication individuals were under age four. Overall, 16 out of 136 individuals (11.7%) died in the entire cohort and eight of them were either Tandem or Other complex duplication. All of the Tandem and/or Other complex duplication deaths occurred between 12 to 23 years, while Translocation and Triplication deaths were 10 or under. We also investigated the cause of death (Column J of Additional file: Table S1) in these individuals. Cause of death was related to the combination of poorly controlled seizures and frequent respiratory infections in seven out of 12 individuals. One individual (BAB11979) died of recurrent lung infections without seizure and one individual (BAB15677) died due to dehydration/septic shock. In the Triplication group, the etiology of death was identified in three individuals and all three individuals died due to discontinuation of supportive care (i.e., ventilator support) given the gravity of their disease which is different than the remaining groups.

### Gene expression and dosage analyses

**Fig. 4:**
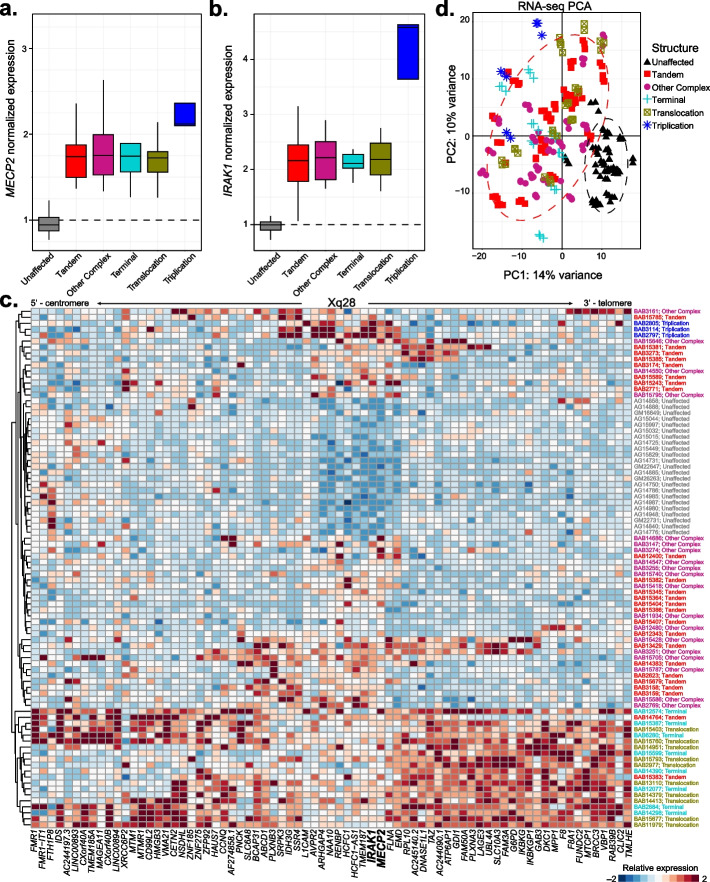
Transcriptomic heterogeneity amongst MRXSL patient-derived lymphoblastoid cell lines. RNA from lymphoblasts from 64 MRXSL individuals from the clinical cohort was collected and processed for RNA-sequencing transcriptomic analyses. Patient lines were collected as triplicate RNA preparations. **a** Normalized *MECP2* expression from RNA-sequencing data. **b** Normalized *IRAK1* expression from RNA-sequencing data. **c** Gene expression along Xq28. Expressed genes are ordered from more centromeric (left) to telomeric (right) within the maximal genomic region spanning the cohort of samples collected. Samples are scaled by column, and rows are clustered using hierarchical clustering using Euclidean distance. **d** Principal component representation of global gene expression changes between MRXSL and unaffected control lines

To assess the impact of the heterogeneity of the genomic rearrangements on the molecular profile of a given patient’s cell, we performed RNA-sequencing on LCLs and fibroblast cells derived from a subset of our cohort (*N* = 64 LCL [24 Tandem, 19 Other complex, 8 Terminal, 10 Translocation, and 3 Triplication] and 18 fibroblast lines [9 Tandem, 6 Other complex, 2 Terminal, and 1 Translocation]). First, we evaluated expression of the SRO genes, *MECP2* and *IRAK1*, between the clinical groups. We observed that *MECP2* expression is indeed increased in MRXSL LCLs regardless of rearrangement type compared to unaffected control LCLs. Importantly, *MECP2* expression is significantly (FDR < 0.1) increased in Triplication LCLs compared to Tandem, Other complex, Terminal, and Translocation rearrangements (Fig. 4a and Additional file: Table S9). *IRAK1* expression is likewise increased in MRXSL LCL regardless of rearrangement type compared to unaffected control LCLs. *IRAK1* is included in the triplicated region, and we remarkably observed an increased expression of *IRAK1* (FDR < 1 × 10^–10^) in triplication lines compared to other MRXSL rearrangements (Fig. 4b). These data suggest that gene expression correlates tightly with copy number supporting the gene dosage hypothesis.

We then asked if correlation with copy number persisted for additional genes on Xq28 outside the SRO, i.e., *MECP2* and *IRAK1*. We clustered gene expression of the detected genes within the genomic coordinates encompassing the entire cohort of measured samples. We found that gene expression in Terminal and Translocation rearrangements clustered separately from the remainder of the cohort (Fig. 4c). Additionally, the Triplication individuals clustered separately as well, while the Tandem and Other complex rearrangement samples were intermixed. We identified a few notable exceptions, for example BAB3161, harboring a complex DUP-NML-DUP/INV rearrangement clustered by itself. The genes distal to the *MECP2* gene were all expressed at a higher level (*F8* through *TMLHE*). We observed a similar phenomenon in BAB12480, which is a complex DUP-NML-DUP/INV rearrangement with a smaller second duplicated segment than BAB3161. Indeed, the genes with the second duplicated segment of BAB12480 were overexpressed (*GAB3* through *FUNDC2*), but not genes mapping to a phased control set just outside of the second duplicated segment like *MTCP1* (Fig. 4c).

Next, we asked if these patterns of gene expression were consistent in an additional cell type by culturing patient-derived fibroblasts. We observed an increased expression of both *MECP2* and *IRAK1* in the MRXSL patient lines, though we were unable to obtain fibroblasts from an individual with a *MECP2* triplication. Like in LCLs, *MECP2* and *IRAK1* expression is increased in MRXSL individuals compared to unaffected controls (Additional file: Fig S3a, b and Table S10). On a patient-by-patient basis, we also found gene expression along Xq28 ordered by the genes in the rearrangement. For example, two siblings (BAB3274 and BAB3275) share the same genomic rearrangement, and these two gene expression measurements clustered together. Furthermore, these patients harbor a triplication and both cell lines have higher expression of the genes within the triplicated region (*e.g.*, *FLNA*) (Additional file: Fig S3c). Taken together, these data demonstrate that genes contained within a region altered by copy number gain increase in expression in multiple cell types.

**Fig. 5:**
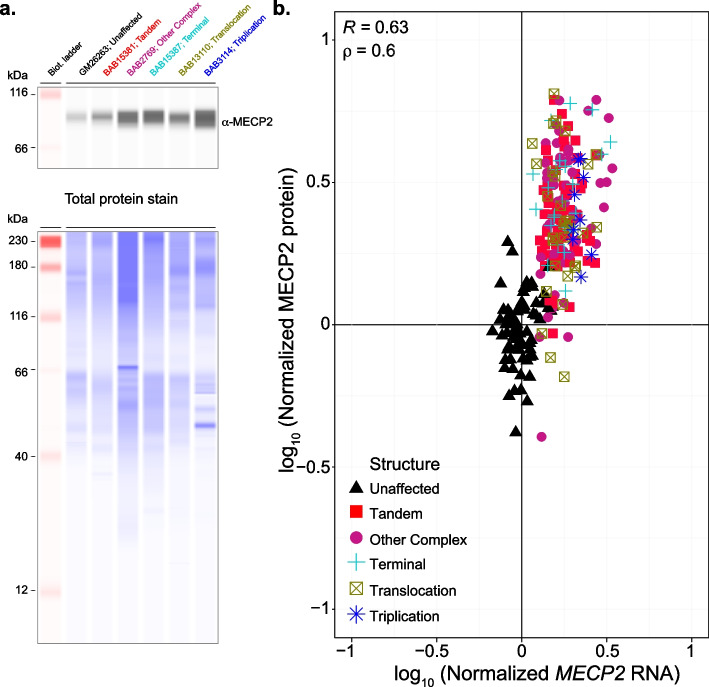
Correlation between *MECP2* RNA and MECP2 protein measurements in patient LCLs. **a** MECP2 protein measurements were made using capillary electrophoresis from matched lysates as collected for RNA-sequencing. MECP2 signal intensity was measured using a Jess Western instrument and normalized to total protein. **b** Correlation between *MECP2* RNA and MECP2 protein; Pearson and Spearman correlation coefficients are displayed in upper left

Given that MECP2 globally regulates gene expression in the brain, we next sought to determine if the global pattern of gene expression was altered in MRXSL individuals compared to unaffected controls. Like the gene dysregulation observed in the brain, we identified thousands of low-magnitude gene expression alterations between MRXSL and unaffected control cell lines. This is reflected by a global separation between genotypes in PCA space in both cell types (Fig. 4d and Additional file: Fig S3d). Lastly, as subtle changes to MECP2 protein levels causes neurological dysfunction [47, 49], we measured MECP2 protein levels matched to the RNA-sequenced samples using quantitative capillary electrophoresis. We first observed that MECP2 protein levels were elevated in MRXSL patient cells. Next, we observed a significant (*p* < 0.05) correlation (Pearson *R* = 0.6, Spearman *p* = 0.63) between the log-transformed *MECP2* RNA levels and MECP2 protein levels (Fig. 5). All together, these data demonstrate that genomic aberrations spanning *MECP2* lead to altered *MECP2* RNA and MECP2 protein levels, leading to global transcriptional dysregulation in MRXSL patient cells.

### Quantitative clinical phenotyping

**Fig. 6:**
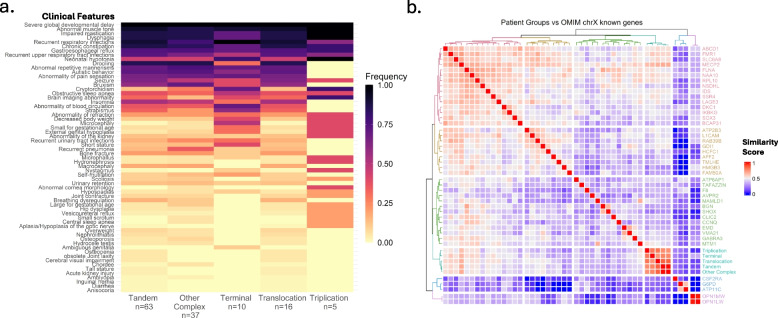
Phenotypic analysis of five different structural variant groups of MRXSL and quantitative similarity analysis of MRXSL features with known OMIM genes on the p and q terminals of X-chromosome. **a** Prevalence of certain features ranged from 0 (light yellow) to 1.0 (black). Individuals carrying Tandem Duplication and Other complex duplication, Terminal duplication and Translocation have similar patterns. Individuals with *MECP2* triplication pattern is different than the other four groups. The scale is provided on the right. DUP: Duplication, TRP: Triplication. **b** The heatmap analysis showed that the highest overlap is among the different structural variants of MRXSL, followed by *MECP2*, *FMR1*, *SLC6A8*, *NAA10* and *FLNA*. Of note, six individuals were excluded from HPO analysis since there were either too little or too many HPO terms

Similar to what was identified in the clinical severity, developmental skills and survival, there were patterned differences in the heatmap of phenotype clustering as captured by HPO analyses. Out of all five groups, subjects with *MECP2* triplication had a distinct pattern compared to other groups. On the other hand, there were clear distinction in phenotypic patterns between Tandem duplication-Other complex duplication vs. Terminal duplication-Translocation group. In the Triplication group, some clinical features such as high pain tolerance, bruxism, drooling, stereotypies, self-mutilation, musculoskeletal anomalies were not observed as several of these features evolve over time. However, some features such as genitourinary anomalies, eye findings and brain imaging abnormalities are more common/severe in the Triplication group (Fig. 6a). Overall, these findings support our clinical observations.

When we compare clinical features of our cohort with Xp and Xq terminals’ disease-causing genes, the highest phenotypic similarity was with the *MECP2* gene as expected. The other genes with relatively high similarity included *FMR1*, *SLC6A8*, *FLNA* and *NAA10* (Fig. 6b). Amongst these genes, *SLC6A8*, *FLNA* and *NAA10* are located in the Xq28 region, whereas *FMR1* is further proximal and locates in the Xq27.3 region. Taken together, our data demonstrate rearrangement class specific phenotypes that most strongly associate with phenotypes driven by *MECP2*.

## Discussion

We performed comprehensive clinical characterization and extensive genomic studies on 137 MRXSL individuals from 125 families (BAB12190 had partial *MECP2* duplication thus excluded from clinical studies). Our study revealed several clinical differences between different genomic rearrangement subgroups of MRXSL with a worsening severity in the order of Tandem duplication < Other complex duplication < Terminal duplication < Translocation < Triplication. This overall severity of phenotype increase was found in DQ, highest achieved gross and fine motor skills, birth weight, postnatal complications/NICU admission, neonatal hypotonia, urinary tract infections, microcephaly, requirement of tube feeding, genitourinary anomalies, movement disorders, eye findings and survival rate. Even for the clinical features which do not show statistically significant difference, the prevalence of the features was either too common (*e.g.*, pneumonia, URI, dysmorphism, chewing/swallowing difficulty, constipation, GERD, abnormal tone, neurobehavioral disorders, dysautonomia, bruxism, high pain tolerance), too rare (*e.g.*, hearing deficits, visual problems) for the MRXSL population, or occurred in too few MRXSL individuals (*e.g.*, sleep apnea).

This is the first study dividing the genomics of MRXSL into categories based on structural variants and investigating these categories’ impact of genomotype on the interindividual variability of phenotype. Our study showed that individuals with *MECP2* triplication (*N* = 5) have the most severe phenotype with significantly decreased survival. Four out of five of the *MECP2* triplication individuals died at 3 weeks, 4 months, 3 years 10 months, and 5 years. The only surviving individual with *MECP2* triplication was at 2 years 9 months at the time of enrollment, and he was ventilator/tracheostomy and G-tube dependent. Only a few cases were reported to have *MECP2* triplication syndrome. Wax et al. reported a fetus with hydronephrosis, fluid-filled bowels, mild ventriculomegaly, and prenasal and prefrontal skin thickening. Patient was born at 30 weeks of gestation and died at 4 weeks of life. [50] Del Gaudio et al. also reported an individual with *MECP2 *triplication (confirmed by fluorescent in situ hybridization (FISH) in addition to CMA) presenting with a more severe phenotype compared to MRXSL individuals in their cohort [16]. Subject had tracheomalacia, hydronephrosis and seizures at 3 months of age. The authors did not report on the survival status of the patient. Tang et al. reported three brothers with *MECP2 *triplication based on multiplex ligation-dependent probe amplification (MLPA) assay [51]. The authors did not perform FISH or other molecular methods to confirm the triplication. Two of the siblings died at 15 and 16 years old, respectively, which is unusual for individuals carrying *MECP2* triplication. However, it is difficult to confirm *MECP2* triplication since MLPA probes may not have interrogated the entire *MECP2* gene or a confirmatory second method has not been conducted. Although critical for families with MRXSL, detecting *MECP2* triplications and distinguishing them from duplications can be challenging in a clinical diagnostic setting. Importantly, most individuals carrying DUP-TRP/INV-DUP present with clinical features similar to Tandem duplication individuals except triplications spanning *MECP2*, which supports the hypothesis that the main gene driving the MRXSL phenotype and contributing to severity is *MECP2*. In our study, *MECP2* gene dosage has been apodictically confirmed by increased expression of the *MECP2 *transcript and MECP2 protein in cell lines from patients with triplication compared to other rearrangement types [52]. It is important to further explore the cascade effects of gene dosage on neurodevelopment versus neurodegeneration regarding downstream effectors of disease phenotype.

The terminal duplication translocation group (*N* = 17) is the second most severe phenotype in MRXSL. Among the 17 subjects with Translocation, 10 of them were translocated to chromosome Y. Pascual-Alonso et al. studied MRXSL in 19 male patients from Spain. Three of those individuals had translocation (two out of three to chromosome Y and one autosome) and additional three individuals had Xp deletion suggesting a recombinant chromosome structure. Although the cohort was small, authors found that neonatal hypotonia, seizure, impaired social interaction and constipation/GERD are more common in the translocation and Xp del MRXSL individuals. Constipation and pain/temperature hyposensitivity were more common in the Translocation group, whereas Xp del MRXSL individuals had seizures more frequently, stereotypical behavior, poor eye contact, impaired social interaction and recurrent infections. The authors concluded that Translocation group is more severe than Xp del group. Our study clearly showed that Translocation group is more severely affected than patients with Terminal duplication, Other complex duplication and Tandem duplication groups. We hypothesize that deletion/duplications of dosage sensitive genes in the autosomal translocated regions can explain the more severe phenotype, however majority of translocations included Y chromosome. Since there are no known neurodevelopmental genes on Y chromosome, we do not currently have an explanation why translocations to chromosome Y are more severely affected. One can hypothesize that neurodevelopmental dosage sensitive genes distal to *MECP2* may contribute to more severe phenotype. However, our study showed that Translocations are more severe than Terminal duplication individuals, thus additional DD/ID genes distal to the *MECP2* cannot solely explain the more severe phenotype in the Translocation group.

Terminal duplications without translocation (*N* = 11) were the third most severe MRXSL group despite we did not see the same tendency regarding the survival rate. However, eight of those individuals were younger than 10 years old (six of whom were younger than four years old or below) thus this survival rate may not represent the actual survival rate as our cohort may represent a selected population causing bias in survival rate. There are few DD/ID genes distal to *MECP2* at Xq28 locus including *L1CAM*, *FLNA*, *GDI1* and *RAB39B*. Duplications of *RAB39B* and *GDI1* are known to cause DD/ID [53, 54]. Our molecular analysis demonstrated that duplications of these distal genes causes increase in their expression. Peters et al. performed genotype–phenotype comparison study on 43 male and 5 female MRXSL individuals using Rett syndrome-specific clinical severity scale (CSS) [13]. The authors identified that duplications including *RAB39B* have a higher CSS score. The worsening severity in their paper can be attributed to Translocation and/or Terminal duplication rather than *RAB39B*. Our molecular profiling provides a dataset to begin to identify risk genes. Given that Translocation and Terminal duplications had an increased frequency of microcephaly, despite normocephaly being a more common MRXSL phenotype, the genes in these distal regions may drive these phenotypes. For example, BAB2627 harbors a tandem rearrangement and BAB3161 harbors a complex rearrangement, both structures encompass genes along the distal portion of Xq28 that are increased in expression. Interestingly, both individuals also had microcephaly. Our study provides a rich dataset for further gene studies and therapeutic investigational studies. Of note, subjects with recombinant X-chromosome resulting in the deletion of *SHOX* gene have the additional clinical feature of short stature as part of the phenotypic spectrum. Recombinant X-chromosome structure should be considered in MRXSL individuals with disproportionate short stature.

Other complex duplication (*N* = 37) and Tandem duplications (*N* = 67) composed the largest groups in the MRXSL population. Overall, these groups were similar in the severity of symptoms. DQs and highest achieved gross motor and fine motor skills were even higher in the Other complex group. Amongst the statistically different clinical features, the rate of normal birth weight, congenital hypotonia, tube feeding, movement anomalies were very similar between these two groups. The difference was observed in postnatal complications, UTI, microcephaly, CAKUT and hearing deficit. Of note, comprehensive genomic studies are needed to resolve tandem duplication and complex rearrangements. For example, BAB15795 seemed to have a tandem duplication on aCGH and optical mapping revealed an inverted duplication. BAB2620 carries an apparent tandem duplication based on aCGH, but genome sequencing, and optical mapping revealed an insertional translocation. BAB3224 and BAB3225 siblings were found to have DUP-TRP/INV-DUP, and BAB11934 and BAB15787 were determined to have DUP-NML-DUP instead of a tandem duplication as suggested by aCGH results.

Interestingly, the mechanisms of formation of Tandem duplication and Other complex duplications are different in most cases. Of note, Other complex duplication group is heterogeneous and includes various types of duplications including but not limited to DUP-NML-DUP, DUP-TRP-DUP, DUP-NML-DUP-NML-DUP and inversions. As we gather further cases, we may have further genomic classifications in the future.

In addition to the differences in the frequency of symptoms between different genomic subgroups, there are qualitative differences. For example, the presence of epilepsy ranged between 40% in the Triplication group to 59% in the Tandem duplication group, which is similar to the literature findings of 43% to 59% in different population studies [7, 9, 55]. Epilepsy is highly relevant in MRXSL because it is the major cause of regression [27, 55] and the most important clinical feature for which families seek treatment [48]. We had previously shown that epilepsy is a dynamic clinical feature and becomes almost universal with age [48]. Thus, considering frequency is not a proper way to distinguish between different groups. However, age of onset gradually occurs earlier as the complexity increases from Tandem duplication to Triplication. Another example is the chewing and swallowing difficulties. Although chewing and swallowing difficulties are highly prevalent in all groups (over 80%), tube feeding requirement gradually increases from Tandem duplication to Triplication. Similarly, the types of visual anomaly and genitourinary problem are more serious in the Triplication individuals.

Lastly, we had one individual who carries a partial duplication of *MECP2* involving the first two exons of *MECP2*. Patient is a 12-year-old male who had undergone chromosomal microarray study due to Myasthenia Gravis symptoms. Patient had no symptoms except dyslexia and difficulty in math. Hanchard et al. reported a family (father and daughter) in whom the entire *MECP2 *is duplicated except the 3’ untranslated region (UTR) [56]. Both individuals had epilepsy and mild cognitive impairment, but father was maintaining an independent life. Thus, the authors concluded that 3-UTR is also required to have the full spectrum of MRXSL phenotype. Moreover, we have individuals with partial *MECP2* triplication (BAB3147 and BAB15420) in our cohort and the clinical course of those individuals align more with Other complex duplication individuals. Our study further validated that partial duplications are not causing an MRXSL phenotype and *MECP2* is the main gene driving the phenotype in Xq28 region.

Our deep profiling of patient cells revealed that duplications spanning *MECP2* drive increased *MECP2* RNA and MECP2 protein expression. Interestingly, while most duplication individuals expressed nearly two-fold protein and RNA, we identified a few duplication individuals with higher than two-fold expression of *MECP2*. These results underscore establishing baseline *MECP2* expression per individual before starting a therapy to reduce *MECP2* expression (*e.g.*, ASO therapy) as these individuals may require higher dosage [57, 58]. Our dataset also provides an opportunity for future work to identify biomarkers that reflect *MECP2* expression or genomic aberration subtype. These results will help to stratify MRXSL individuals for personalized therapies.

## Conclusions

In conclusion, genomic studies on a large cohort of MRXSL and comparison with comprehensive clinical profile showed that the clinical severity gradually worsens from Tandem duplication to Triplication not only due to increase in the prevalence of symptoms, but also due to severity and timing of symptoms such as age of epilepsy onset, tube feeding, CAKUT, visual problems. *MECP2* is the major disease contributing gene since its dosage and the structure of CNV drive the phenotype in Xq28 duplication individuals.

## Supplementary Information

Additional File 1.

Additional File 2: Additional files include three supplemental figures. Fig S1 explains the workflow, Fig S2 includes the entire cohort’s CNV size and gene content, and Fig S3 shows the transcriptome profile between genomic subgroups in fibroblast.

## Data Availability

Microarray data generated in this study are available through GEO [59] under the accession numbers, GSE49447 [60] and GSE250451 [61]. New data can be found in GEO accession GSE256075 [62]. At the time of publication there is no access-controlled repository for Optical Genome Mapping (OGM) data therefore OGM data in this study is available on reasonable request. BAM files from individuals who consented to share data in public controlled datasets are available in dbGaP [63, 64] study phs002999.v4.p1 [65] and SRA [66] BioProject PRJNA953021. Breakpoints detected by Oxford Nanopore minion sequencing can be found in SRA BioProject PRJNA1169102 [67] Raw RNA-sequencing files from probands and unaffected controls who consented to share data in public controlled datasets are available in dbGaP study phs002999.v4.p1.

## Abbreviations

aCGH: Array Comparative Genomic Hybridization
ASD: Autism Spectrum Disorder
CA: Chronological Age
CAKUT: Congenital Anomalies of the Kidney and Urinary Tract
CGR: Complex Genomic Rearrangements
CNV: Copy Number Variants
CSS: Clinical Severity Scale
ddPCR: Droplet digital PCR
DD/ID: Developmental Delay/Intellectual Disability
DUP-NML-DUP: Duplication-Normal-Duplication
DUP-TRP/INV-DUP: Duplication-Triplication (Inverted)-Duplication
DQ: Developmental Quotient
FISH: Fluorescent in Situ Hybridization
GERD: Gastroesophageal Reflux Disorder
GI: Gastrointestinal
GU: Genitourinary
HPO: Human Phenotype Ontology
LCL: Lymphoblastoid Cell Line
MECP2: Methyl CpG Binding Protein 2
MRXSL: X-linked intellectual developmental disorder Lubs type
ONT: Oxford Nanopore Technologies
SRO: Smallest Region of Overlap
SV: Structural Variant
UHMW: Ultra-High Molecular Weight
UTI: Urinary Tract Infections
WGS: Whole Genome Sequencing
