# Supplementary material for: Structural variant allelic heterogeneity in MECP2 duplication syndrome provides insight into clinical severity and variability of disease expression
Source: Genome Med. 2024 Dec 18;16:146. doi: 10.1186/s13073-024-01411-7 (PMC11658439; doi:10.1186/s13073-024-01411-7)
Supplement: Supplementary file 1 — Additional File 1. [file 13073_2024_1411_MOESM1_ESM.zip › Table S3_R1.docx]

**Table S3: ddPCR primers and probes applied to this study.**

| **Primer name** | **5’-Primer Sequence-3’** | **GRCH37 Coordinates** |
| --- | --- | --- |
| MECP2CNV_FWD | 5’-TCCTTTGCTTAAGCTTCCGT-3’ | chrX:153297701-153297720 |
| MECP2CNV_REV | 5’-GCAGAGACATCAGAAGGGTC-3’ | chrX:153297832-153297851 |
| MECP2CNV_ProbeFAM | 5’-[6FAM]-CTTCCGGCACAGCCGGGGCG-[BHQ1] -3’ | chrX:153297806-153297825 |
| RPP30_FWD | 5’-GATTTGGACCTGCGAGCG-3’ | chr10:92631759-92631776 |
| RPP30_RWD | 5’-GCGGCTGTCTCCACAAGT-3’ | chr10:92631803-92631820 |
| RPP30_ProbeHEX | 5’-[HEX]- CTGACCTGAAGGCTCT-[BHQ1] -3’ | chr10:92631781-92631796 |

ddPCR: Droplet digital PCR; GRCH37: Genome Research Consortium Human Build 37
