# Supplementary material for: Structural variant allelic heterogeneity in MECP2 duplication syndrome provides insight into clinical severity and variability of disease expression
Source: Genome Med. 2024 Dec 18;16:146. doi: 10.1186/s13073-024-01411-7 (PMC11658439; doi:10.1186/s13073-024-01411-7)
Supplement: Supplementary file 1 — Additional File 1. [file 13073_2024_1411_MOESM1_ESM.zip › Table S4_ESM.docx]

**Table S4: Coordinates for Minion Sample Enrichment (T2T)**

| Sample | Coordinates |
| --- | --- |
| BAB15787 | chrX:151129558-1535666  chrX:1384311-3584311 |
| BAB15785 | chrX:150996957-153531400 |
| BAB15790 | chrX:151016327-152994525 |
| BAB15795 | chrX:151471239-153427784 |
| BAB15793 | chrX:151343776-152743776  chrX:1-600000 |
| BAB11934 | chrX:89040020-90259856  chrX:152565192-53919869  chrY:3085649-4556488 |
| BAB15760 | chrY:18319460-62460029  chrX:144060309-146060309 |
| BAB14606 | chrX:151139107-152875069 |
| BAB3224 | chrX:150040754-153490915 |
