# Supplementary material for: Structural variant allelic heterogeneity in MECP2 duplication syndrome provides insight into clinical severity and variability of disease expression
Source: Genome Med. 2024 Dec 18;16:146. doi: 10.1186/s13073-024-01411-7 (PMC11658439; doi:10.1186/s13073-024-01411-7)
Supplement: Supplementary file 1 — Additional File 1. [file 13073_2024_1411_MOESM1_ESM.zip › Table S5_ESM.docx]

**Table S5: Primers to amplify and Sanger sequencing breakpoint junctions.**

| **Name** | **sequence 5'-3'** | **start(hg19)** | **end(hg19)** |
| --- | --- | --- | --- |
| K4R | CTAGGGCTGGGCTAGGTGA | chrX:153564632 | chrX:153564656 |
| K5F | CTCCTGGGTCTTCCTCAGTG | chrX:153163099 | chrX:153163123 |
| BAB3140_jct1_R1 | CCTCCAGCTATGGCCAAACGTGGATAACAGC | chrX:152923093 | chrX:152923123 |
| BAB3140_jct1_R2 | GACAAAGTGACCTGGGATGCAGCG | chrX:153035534 | chrX:153035557 |
| BAB3140_jct2_F1 | ATTAATTCACGGCACCACTGGCAT | chrX:153433216 | chrX:153433239 |
| BAB3140_jct2_F2 | CTCACTGAGCCGTCAATCTGGTTGGC | chrX:153034867 | chrX:153034892 |
| BAB3224_5_jct1F1 | CATTAACCAAGTAAGCCCCTCATCTGTGAAC | chrX:152422376 | chrX:152422406 |
| BAB3224_5_jct1F2 | CTGTGTGTCTCAAAGGTTGGGGGTAG | chrX:151909528 | chrX:151909553 |
| BAB3224_JCT2F1_B | GGACTCCTGGCAGAAAGGGCCAGCCATGCCCAGATGACACTG | chrX:152234057 | chrX:152234098 |
| BAB3224_JCT2F2_B | GGCTTGTTCACTACTCCATGGGTGAGGCCAGGCCCATAGG | chrX:153582321 | chrX:153582360 |
| BAB11934_jct1_R2 | GACTCCCTGACACCTTGGAGTAGGCACTG | chrX:153065031 | chrX:153065059 |
| BAB11934_jct1_R3 | CAAGTGTTAGAGTTTCACCCAAAGCCCTTGACATAG | chrX:89406919 | chrX:89406954 |
| BAB14951_F1 | ATTCTGTGTGGCCTTTTGCATGATAGGTTGGC | chr6:509378 | chrX:509409 |
| BAB14951_R2 | CCTCATACCTTAGATATCCATTGCCCACCCC | chrX:152107766 | chrX:152107796 |
| BAB15760_R1 | TATTTGCCGAGAGCTGTTTATAGTA | chrY:20239290 | chrY:20239314 |
| BAB15760_R2b | CAAAAAATACTTTGTAAAATTGAACTATATCTTTA | chrX:145894100 | chrX:145894134 |
| BAB15785_longR4 | CCTGAACACATCCCTATTTAAATTACGATATTAC | chrX:152988049 | chrX:152988082 |
| BAB15785_longF5 | GCCAGATACATTCCTTGATCTCATACAGCTGACAGTCTACGGCAG | chrX:153479477 | chrX:153479521 |
| BAB15785_sangerR | GATTAGTTTTCCAGGCTGGGCGCGGTG | chrX:152987018 | chrX:152987044 |
| BAB15785_sangerF | CCACTGACAAATGTAAGAATTCAAC | chrX:153483598 | chrX:153483797 |
| BAB15787_Fxp | GCAAGTGTCTGCAATTTCCTCACC | chrX:3172672 | chrX:3172695 |
| BAB15787_Rxq | CGTGGCCCTAGAATAGTGGAGTCCAG | chrX:153128108 | chrX:(153128133) |
| BAB15787_jct2_F3 | CTCAGAATGGCCAGAGTGCTGCCATAAAGCAGGTG | chrX:153554884 | chrX:153554918 |
| BAB15787_jct2_R3 | CACGCGCCTGGCCTACGCTCTCAATAATACTCTTCC | chrX:2678237 | chrX:2678272 |
| BAB15790_F1 | GACTGTAAATGTCATTAGTCCTGCCAGG | chrX:153386115 | chrX:153386142 |
| BAB15790_R1 | GAAGCTTCTGTTTCCTTGTTTGC | chrX:152617494 | chrX:152617516 |
| BAB15793_R1b | TGTCCTCAATGAGACCTAGGCTCAGTGCAG | chrY:115476 | chrY:115505 |
| BAB15793_R2b | CAGGCAGTGGCTGTCCTCCTCATAAC | chrX:153035318 | chrX:153035343 |
| BAB15795_R1 | CTCCCTCTGCCCAAACTGGCTACAG | chrX:153564632 | chrX:153564656 |
| BAB15795_R2 | AATTAGCTGGGTGTGCTGGCGTGCG | chrX:153163099 | chrX:153163123 |
