# Supplementary material for: Structural variant allelic heterogeneity in MECP2 duplication syndrome provides insight into clinical severity and variability of disease expression
Source: Genome Med. 2024 Dec 18;16:146. doi: 10.1186/s13073-024-01411-7 (PMC11658439; doi:10.1186/s13073-024-01411-7)
Supplement: Supplementary file 2 — Additional File 2: Additional files include three supplemental figures. Fig S1 explains the workflow, Fig S2 includes the entire cohort’s CNV size and gene content, and Fig S3 shows the transcriptome profile between genomic subgroups in fibroblast. [file 13073_2024_1411_MOESM2_ESM.pdf]

Fig S1. Workflow of the clinical and genomic studies. All MRXLS probands underwent custom-designed high-resolution aCGH which has a 1 probe/800 bp resolution. Then all patients underwent short-read GS (37X coverage). Patients who were found to have CGR on aCGH and GS underwent long-read on either Promethion or MinION Flow cells [ONT (3X-40X coverage) or PacBio HiFi (10X-20X coverage) and/or Optical Mapping. aCGH: array Comparative Genomic Hybridization, bp: base pair, MRXLS: *MECP2* Duplication Syndrome, GS: Genome Sequencing, ONT: Oxford Nanopore Technologies.

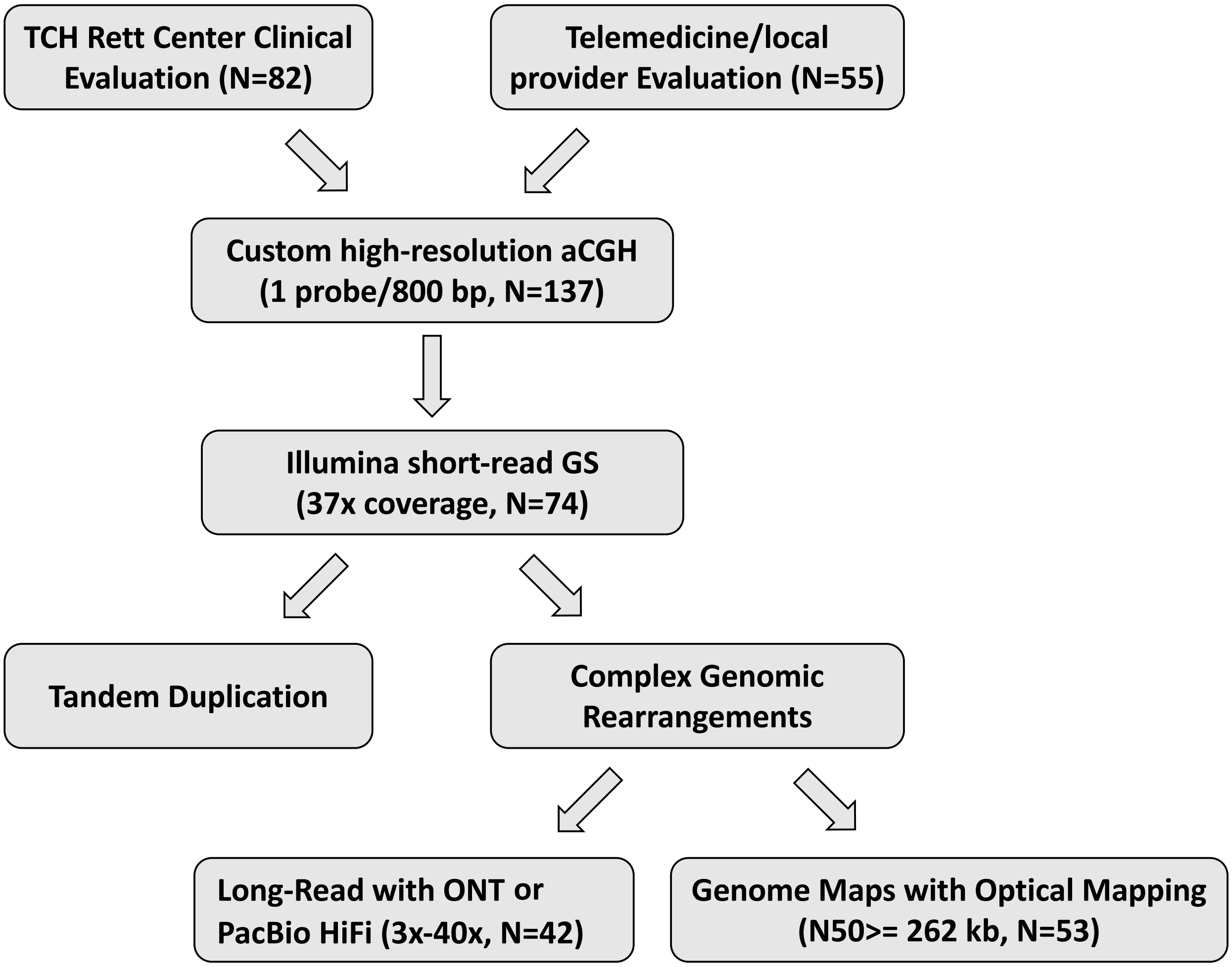

Fig S2: CNV size and gene content across Xq26-Xq28 in 125 unrelated probands. a. Individual CNVs coordinates mapped by custom aCGH are grouped by genomic structure, * indicates apparently simple duplications with structures that are not completely solved. Proband identification is shown on the left. Red bars indicate duplicated region; blue bars indicate triplicated region. Blue arrows indicate *MECP2* is triplicated. Genes shown below are disease causing genes as per OMIM. Smallest Region of Overlap (SRO) is shown as the gray bar **b**. Reported *MECP2* cis-regulatory elements (CRE) are indicated as F11, F16, F17, F21, F3, F13.[1,2] Tandem duplication group includes individuals carrying unsolved duplications as detected by aCGH.

a.

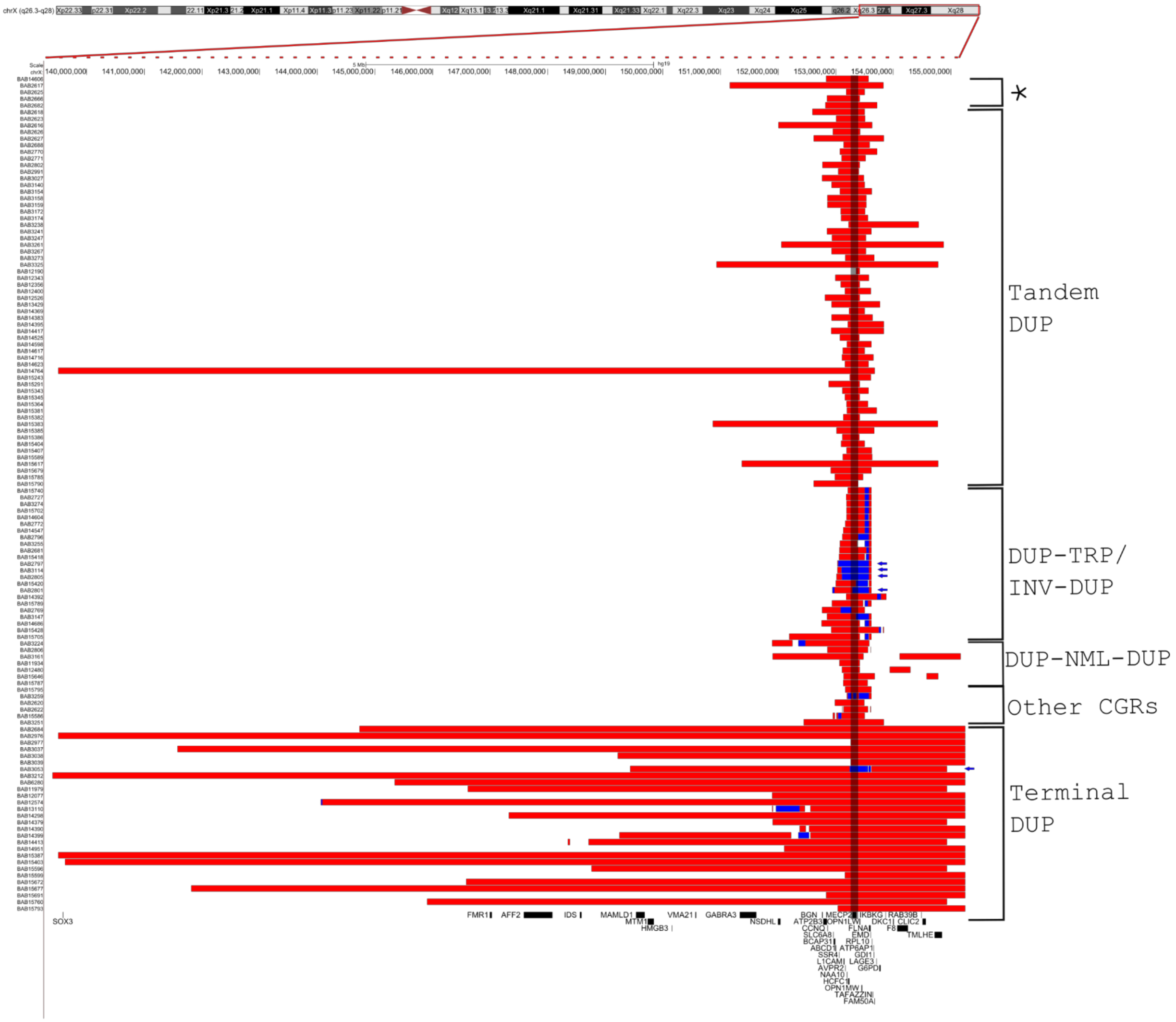
*
Tandem DUP
DUP-TRP/ INV-DUP
DUP-NML-DUP
Other CGRs
Terminal DUP

b.

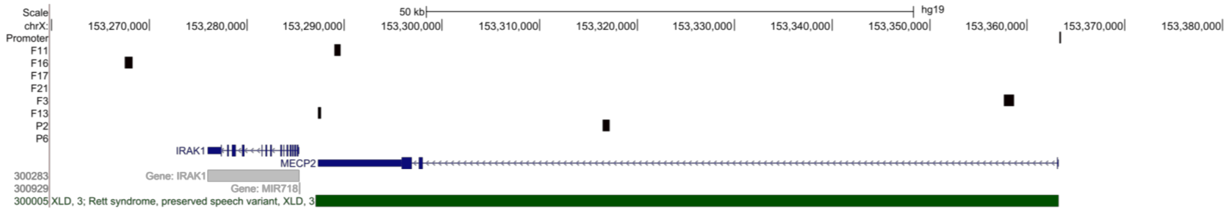
Scale
chrX:
Promoter
F11
F16
F17
F21
F3
F13
P2
P6
50 kb
hg19
153,270,000
153,280,000
153,290,000
153,300,000
153,310,000
153,320,000
153,330,000
153,340,000
153,350,000
153,360,000
153,370,000
153,380,000
IRAK1
MECP2
300283
Gene: IRAK1
300929
Gene: MIR718
300005 XLD, 3; Rett syndrome, preserved speech variant, XLD, 3

Fig S3. Transcriptomic heterogeneity amongst MRXLS patient-derived fibroblast cells. RNA from fibroblasts from 18 MRXLS individuals from the clinical cohort was collected and processed for RNA-sequencing transcriptomic analyses. Patient lines were collected as triplicate RNA preparations. **a.** Normalized *MECP2* expression from RNA-sequencing data. **b.** Normalized *IRAK1* expression from RNA-sequencing data. **c.** Gene expression along Xq28. Expressed genes are ordered from more centromeric (left) to telomeric (right) within the maximal genomic region spanning the cohort of samples collected. Samples are scaled by column, and rows are clustered using hierarchical clustering with Euclidean distance. **d.** Principal component representation of global gene expression changes between MRXLS and unaffected control lines.

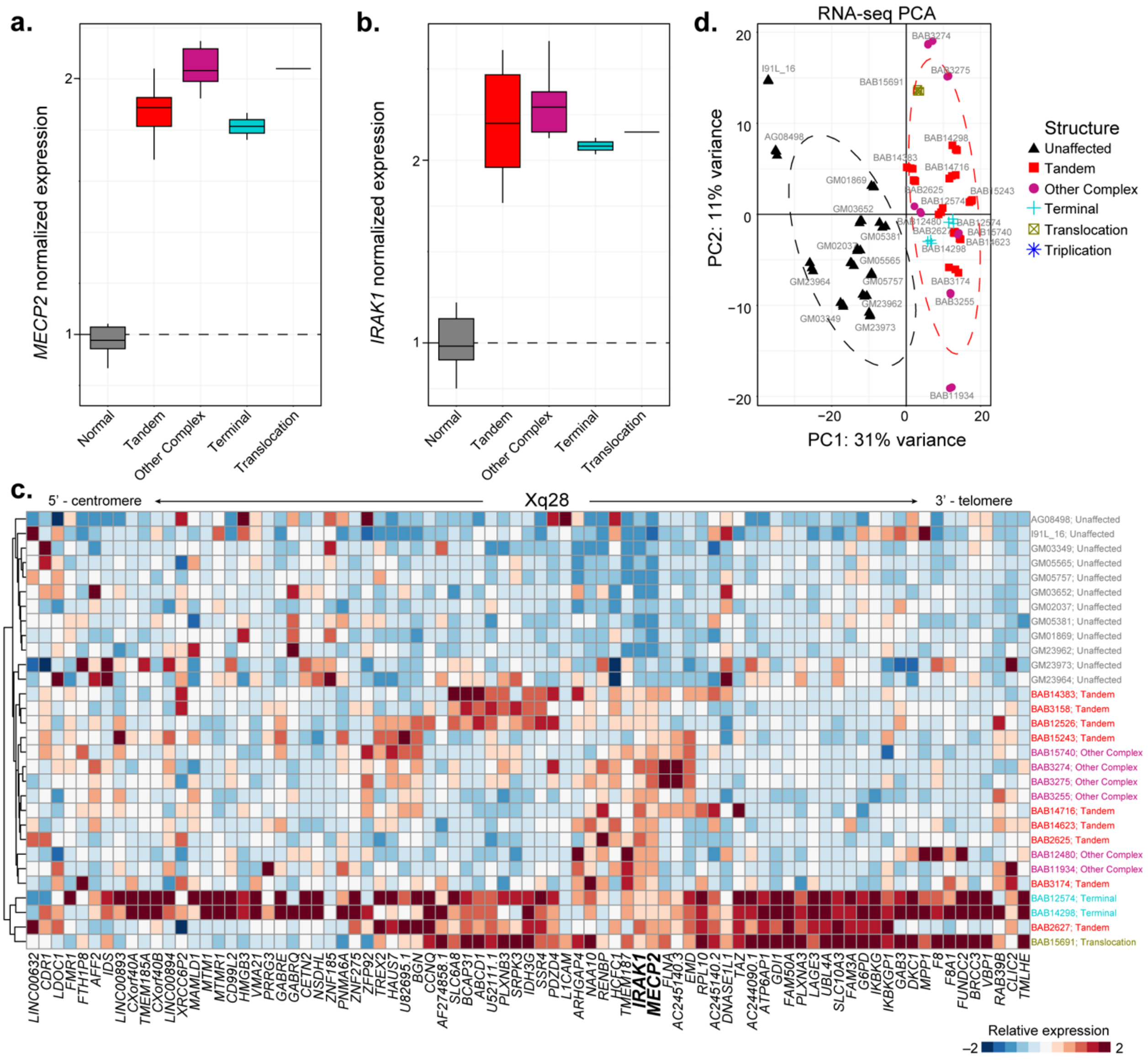

1. Liu, J., and Francke, U. (2006). Identification of cis-regulatory elements for MECP2 expression. Hum Mol Genet *15*, 1769-1782. 10.1093/hmg/ddl099.
2. Shao, Y., Bajikar, S.S., Tirumala, H.P., Gutierrez, M.C., Wythe, J.D., and Zoghbi, H.Y. (2021). Identification and characterization of conserved noncoding cis-regulatory elements that impact Mecp2 expression and neurological functions. Genes Dev *35*, 489-494. 10.1101/gad.345397.120.
